# A review of multicomponent synthesis of Betti-base amidoalkyl naphthol (AAN) derivatives as substances of biological interest

**DOI:** 10.1039/d6ra05415c

**Published:** 2026-10-06

**Authors:** Seyyed Hojjat Sadeghi, Sadegh Rostamnia

**Affiliations:** a Advanced Porous Materials and Energy Technology (APMET) Res. Lab., Department of Chemistry, Iran University of Science and Technology (IUST) Tehran 16846-13114 Iran rostamnia@iust.ac.ir srostamnia@gmail.com

## Abstract

The effective one-pot Mannich-type multicomponent Betti reaction is the primary method for preparing amidoalkyl naphthols (AANs), which are important scaffolds in medicinal chemistry. While this approach has an intrinsic step economy, achieving sustainable synthesis requires a more thorough examination of the catalytic systems. With a special emphasis on quantitative sustainability evaluation, this comprehensive review critically examines current developments in both homogeneous and heterogeneous catalysis for AAN synthesis. We present a rigorous evaluation of several catalytic systems, testing them against quantitative green criteria such as atom economy, recyclability, and energy input, and find that immobilised heterogeneous catalysts have a superior environmental profile to that of typical homogeneous catalysts. The performance differences in terms of catalyst structure and active-site characteristics are explained mechanistically. This review provides important approaches to the synthesis of this benchmarks for synthetic chemists seeking economically feasible and environmentally responsible approaches to this crucial class of molecules.

## Abstract 2

The effective multicomponent Betti reaction for preparing amidoalkyl naphthols (AANs).

## Graphical abstract

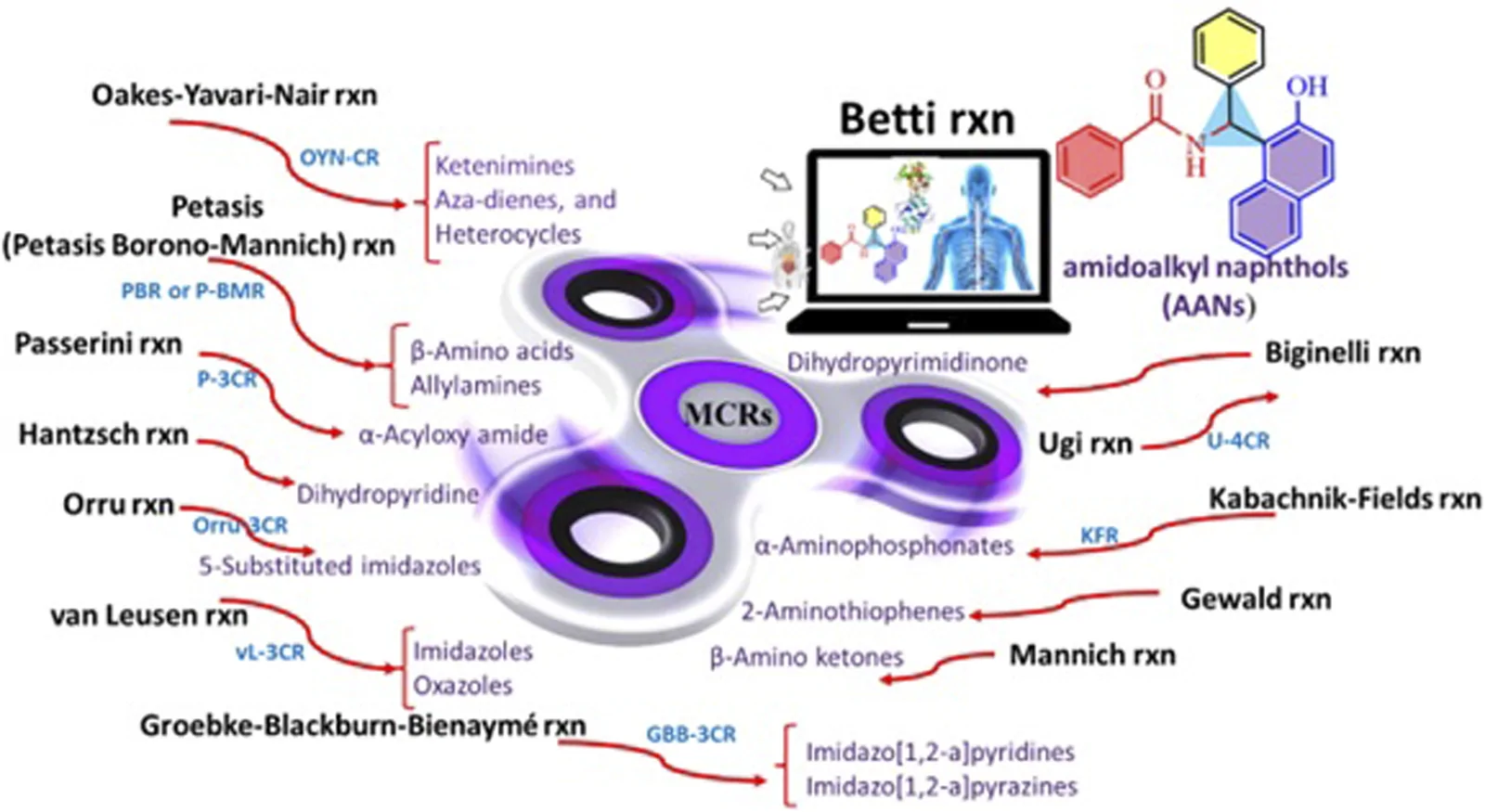

## Introduction

The efficient, single-step formation of complex compounds makes one-pot multicomponent reactions (MCRs), commonly referred to as zipper reactions, an essential synthetic technique in contemporary organic chemistry. This methodology is highly beneficial for synthesizing physiologically significant substances, such as medications. The primary benefit of MCRs is their ability to simultaneously mix multiple starting materials in a single pot, producing new products without the need to separate intermediates. This capability aligns with the principles of green chemistry.^1–4^ Multicomponent reactions (MCRs) have gained significant popularity in modern synthetic organic chemistry because of their ability to form carbon–heteroatom bonds and carbon–carbon bonds. The carbon atoms of all the initial ingredients are essentially contained in one pot.^5–10^

In MCR condensations (Fig. 1), more than three compounds are combined simultaneously in a single pot to carry out a series of sequential reactions to create new products that contain the key components of all the starting materials without isolating intermediate or accessory products. Additionally, multi-step methods used in other sectors cannot compete with these two-component processes, which feature an easy one-pot operation, potential structural changes, the ability to construct complex molecules, increased reaction efficiency, and reduced laboratory operations.^11,12^ The examples of different types of multicomponent reactions, including the Passerini, Ugi, Petasis, Oakes–Yavari–Nair (OYN),^2^ Hantzsch, Orru, van Leusen, Groebke–Blackburn–Bienayme (GBB), Mannich, Gewald, Kabachnik-Fields, Biginelli and Betti reactions, are presented in Fig. 1.

**Fig. 1:**
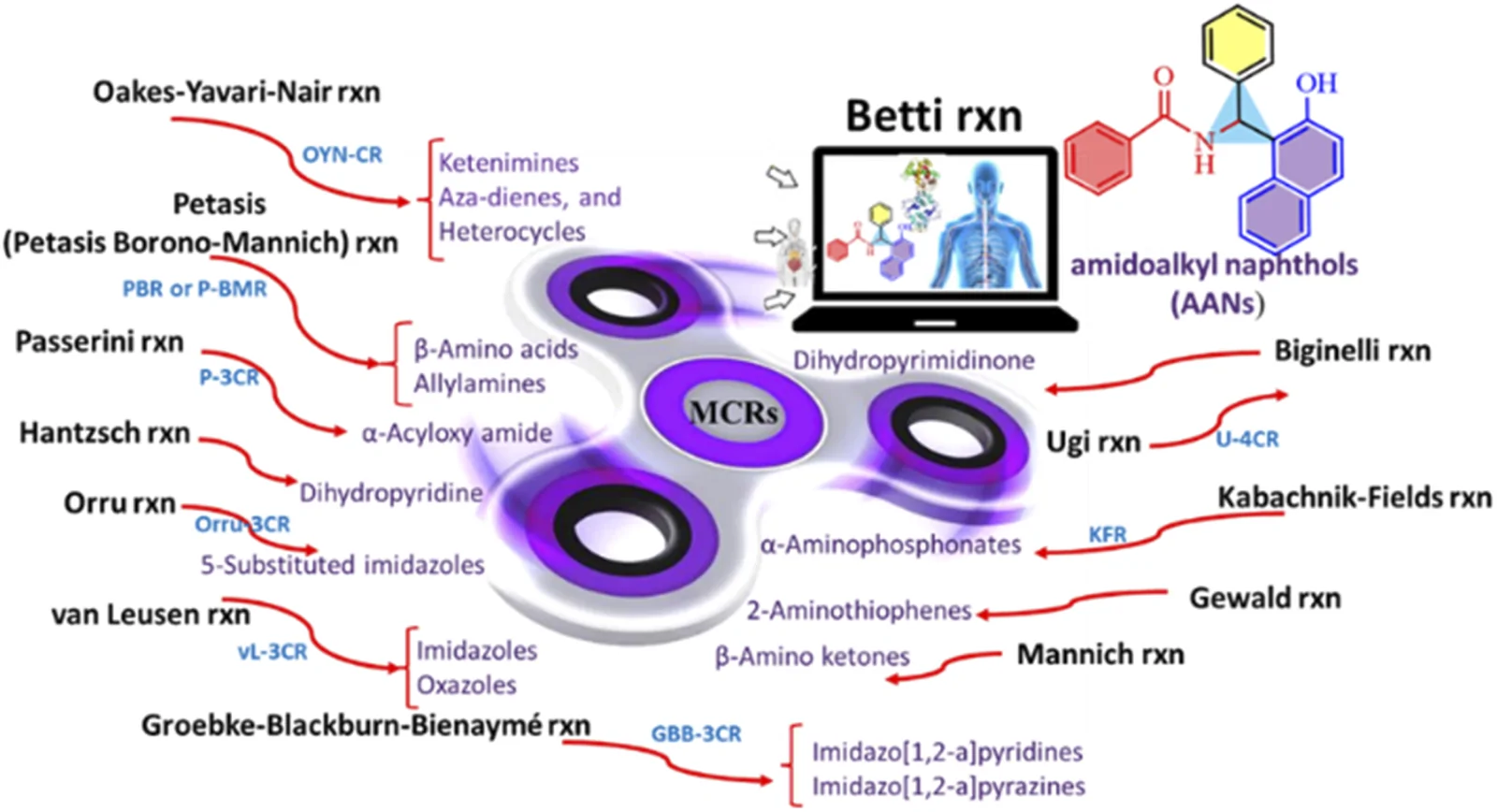
Various multicomponent reactions.

Heterocyclic compounds (HCs) are among the most important compounds in organic chemistry because they constitute the framework of many pharmaceutical and biological substances. HCs are cyclic compounds that contain heteroatoms such as nitrogen, sulfur, and oxygen. Among the various heterocyclic compounds that exist, heterocycles that have a nitrogen atom in their structure are of particular importance.^13–15^ The unique structure of AANs makes them a class of compounds of significant importance in medicinal chemistry, often serving as a framework for various biological and pharmacological substances. These compounds, which are members of the significant Betti base derivative family of N-containing heterocyclic compounds, have been shown to exhibit a range of biological activities, including antibacterial, antioxidant, and antiparasitic properties.^16–20^

Green chemistry, also known as safe or sustainable chemistry, involves the design and development of products and chemical processes that minimize or eliminate the use of compounds detrimental to human health and the environment. Green chemistry is a rapidly developing area of chemistry that has garnered increasing interest since the early 1990s. This emerging field of science supports eco-friendly reaction media, catalytic processes, and the concept of an atom economy that produces little to no waste, as well as bioremediation and modifications to engineering techniques. Health and safety have expanded from small-scale laboratory production to large-scale industrial manufacturing through the use of diverse green chemistry techniques and organic synthesis. Green chemistry started with the Environmental Protection Act of 1990. Instead of treating pollutants once they are created, it focuses on preventing pollution at the source. In 1991, the Environmental Protection Agency (EPA) made this mission statutory. The application of these principles to AAN synthesis focuses primarily on minimizing the environmental impact through two key areas: (1) catalyst sustainability: prioritizing catalysts with high atom efficiency, low toxicity, and, most importantly, high recyclability to minimize catalyst loss and waste generation. (2) Reaction medium: shifting from conventional volatile organic solvents (VOCs) to benign alternatives, such as water or deep eutectic solvents, or, ideally, developing solvent-free mechanochemical or microwave-assisted methods, to drastically reduce energy input and solvent waste^21–23^ (Fig. 2).

**Fig. 2:**
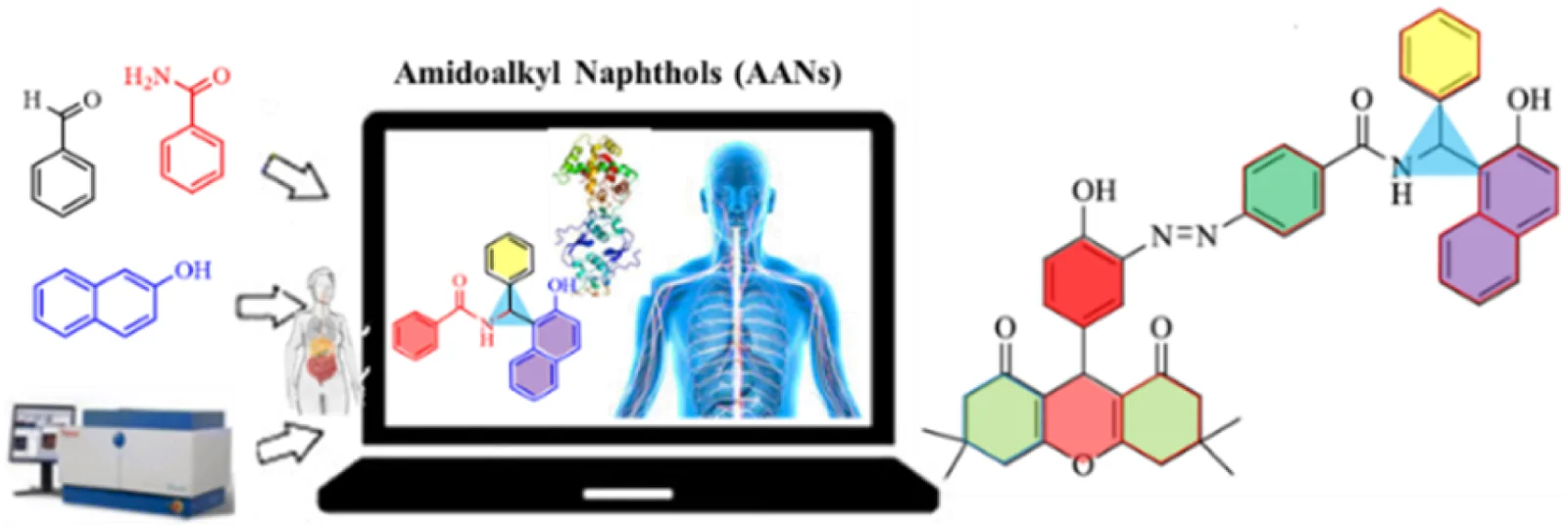
Synthesis of amidoalkyl naphthol derivatives.

Green chemistry refers to the development of chemical compounds and procedures that minimize the usage and production of hazardous compounds.^24–27^ To meet the demands of green chemistry, which aims to reduce or eliminate the production and use of hazardous compounds, the synthesis of AANs has evolved significantly in recent years. The multicomponent Mannich reaction is an environmentally friendly method for producing AANs that can be performed without the use of solvents. The overall goal of developing eco-friendly catalytic processes that increase energy efficiency and reduce waste is in line with this.^28^

The term “Betti base amidoalkyl naphthols (AANs) moieties” refers to a highly significant class of heterocyclic organic compounds that are valued primarily for their versatile applications in medicinal chemistry and asymmetric synthesis. Betti base amidoalkyl naphthols (AANs) are a specific family of functionalized naphthol derivatives named after the chemical reaction used to synthesize them. The carbon atom that links the naphthol ring to the amide nitrogen is often a stereocenter. This chiral nature makes AANs highly relevant as ligands and catalysts in asymmetric synthesis. AANs are most commonly synthesized *via* the Betti reaction, which is an efficient, one-pot, Mannich-type multicomponent reaction (MCR). This reaction involves the condensation of three components.^29–31^ The Betti reaction, which produces amino benzyl phenols from the reaction of aldehydes, phenols, and primary amines, was first introduced at the beginning of the 20th century.^32–34^ This reaction produces amino benzyl naphthols and related derivative chemicals such as amidoalkyl naphthoquinones and amidoalkyl β-naphthols.^35,36^ Quinones are crucial biological components of many pharmaceuticals and natural materials. The importance of naphthoquinones, a class of quinones, can be attributed to their anticancer, antiallergenic, antimalarial, and antibacterial effects.^37–39^ Oxazine derivatives, which are generated from amidoalkyl β-naphthols, are key substances in medical and industrial applications.^40–42^ The hydrolysis of amides can transform amidomethyl β-naphthols into significant pharmacological and biological compounds. Amidoalkyl compounds have been prepared using various heterogeneous and homogeneous catalysts, including basic and acidic catalysts,^41,43–51^ magnetic catalysts,^52–54^ ionic liquids,^55–57^ and metallic nanoparticles.^58–61^

### Amidoalkyls as bioactive molecules

AANs have a range of biological characteristics, including antimicrobial, antiviral, antiparasitic, antifungal, and antitumor activity, as well as anti-U87 cancer cell effects.^62–68^ In addition to being promising antioxidants, these substances were examined as α-glucosidase and cholinesterase inhibitors.^69–72^ AAN derivatives are synthesized from aldehydes, pyrazolyl- and di-indolyl oxazine, and 2-naphthol. Some of the novel compounds show potential antibacterial action, as shown in Fig. 3.^62^ According to the antibacterial test results, compounds 1 and 2 (1Ph and 2Me) exhibited more pronounced inhibitory effects on *Bacillus subtilis* and *Klebsiella pneumonia*, respectively, than on *Staphylococcus aureus*. All four variants exhibited antibacterial properties similar to those of the reference agent gentamicin.

**Fig. 3:**
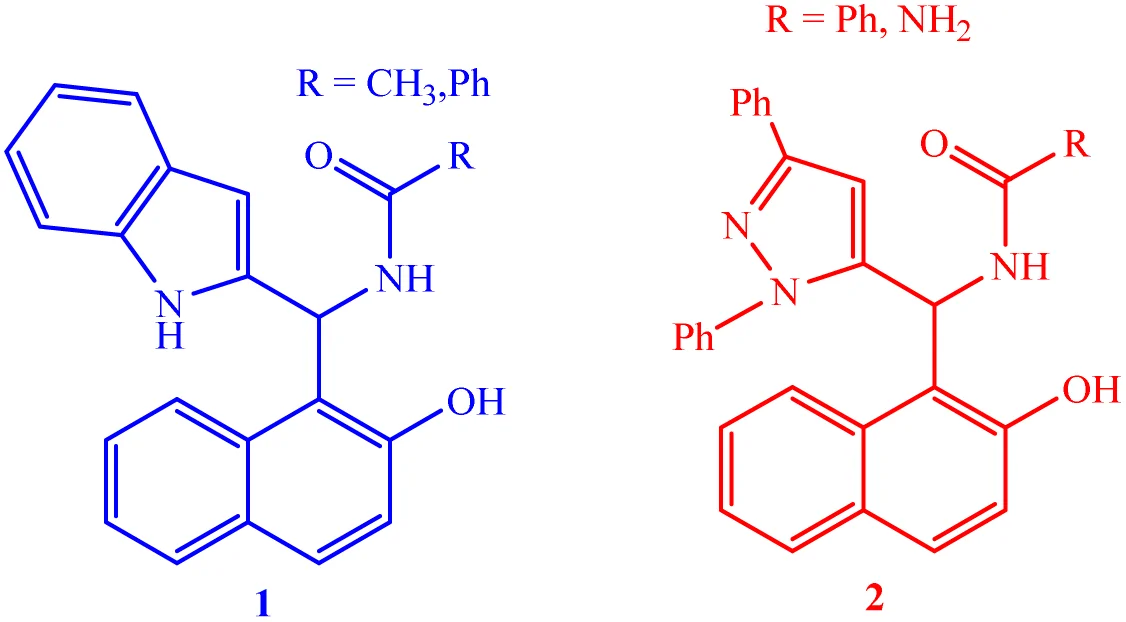
Several typical AAN compounds have antibacterial and antiviral characteristics.

The antibacterial activity of 14 AANs containing an azo fragment was evaluated *in vitro*.^63^ Compared with the reference medications trimethoprim/sulfamethoxazole (SXT) and ciprofloxacin, none of the synthesised derivatives exhibited superior antibacterial properties. In contrast, antifungal tests conducted on compounds 3 and 4 (Fig. 4) revealed that these compounds were more effective against *Aspergillus niger* than the medications miconazole and fluconazole.

**Fig. 4:**
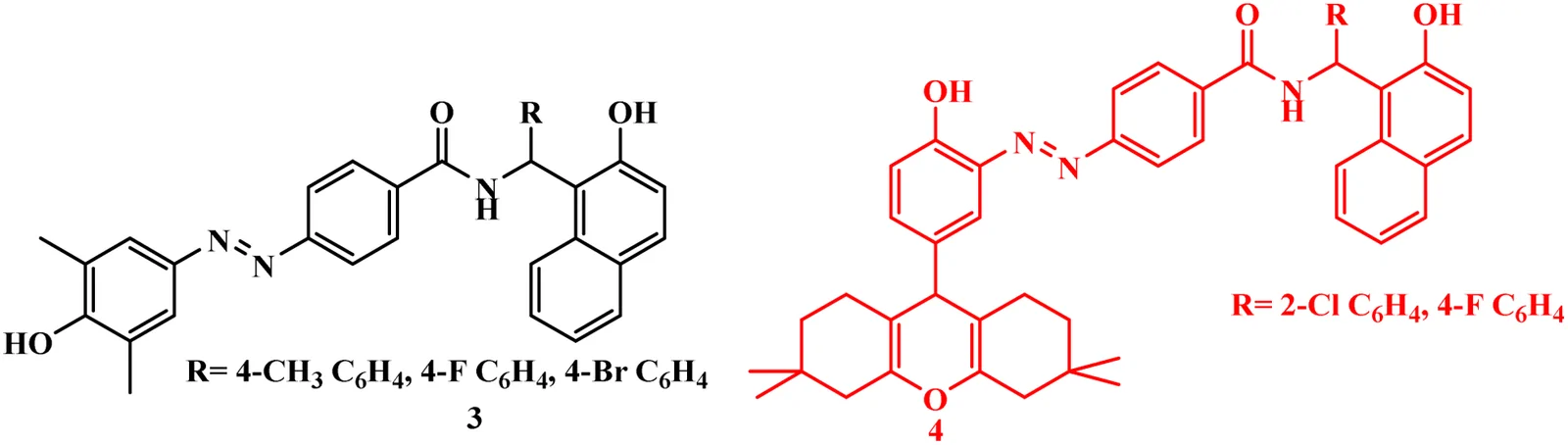
AAN compounds and antifungal agents.

One study reports the ability of AAN to prevent *Helicobacter pylori* infection *in vitro*.^64^*Helicobacter pylori*, the primary cause of duodenal ulcer disease, stomach cancer, and chronic gastritis, is present in more than half of the world's population. Based on the results, AAN derivatives are considered the most effective anti-Helicobacter factors; derivative 5 (Fig. 5) is the most effective.

**Fig. 5:**
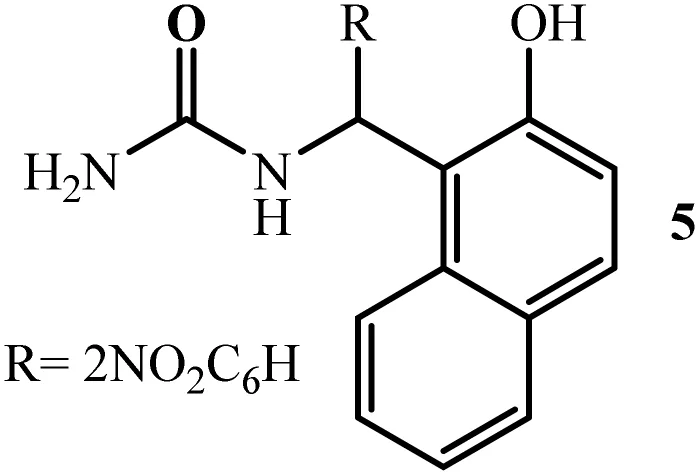
Promising substances of the AAN class that have anti-*Helicobacter pylori* properties.

Various benzaldehydes and long-chain amide derivatives have been used to synthesize various AANs. Their antimicrobial properties were also assessed *in vitro*.^65^ According to these findings, derivative 6 (Fig. 6) exhibits the most effective activity against *Escherichia coli* (ATCC 25 922) and *Staphylococcus aureus* (ATCC 25 923) compared with the reference, gentamicin.

**Fig. 6:**
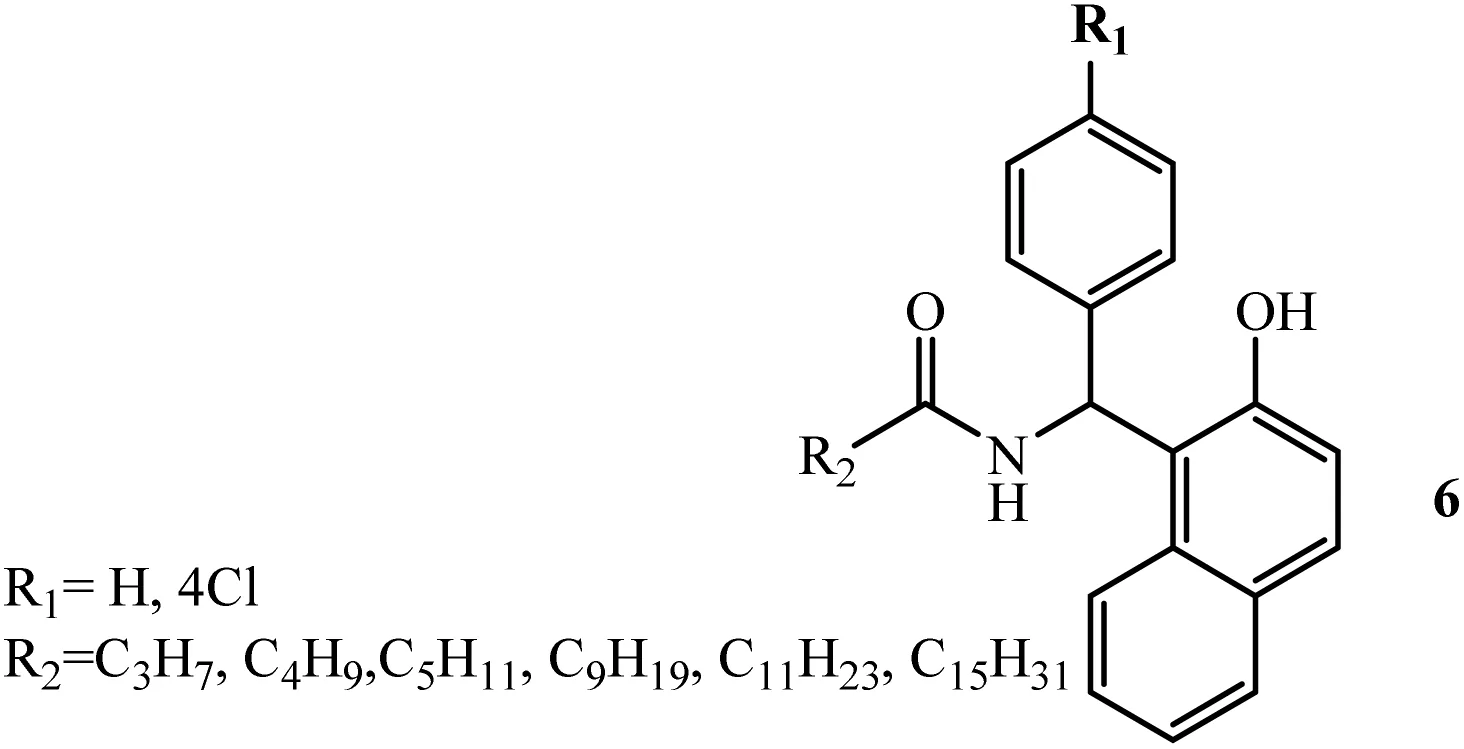
Potent AANs against *Escherichia coli* and *Staphylococcus aureus*.

Leishmania is a parasitic protozoan that seeks to infiltrate and grow within macrophages. Additionally, macrophages are immunological defense cells that phagocytose a variety of microorganisms, including bacteria and fungi. They cannot, however, eradicate this parasite, which lives in the phagosomes of contaminated macrophages and grows there, destroying the infected macrophages and causing Leishmania wounds to appear.^73–75^ This research revealed the antiparasitic properties of AAN derivatives 7.^66^ To assess antileishmanial efficacy against *Leishmania donovani* extracellular promastigotes, all AAN compounds were tested. The four derivatives shown in Fig. 7 demonstrated anti-Leishmania action. Based on molecular docking studies conducted against the Leishmania major pteridine reductase 1 (PTR1) protein (PDB IB 6RXC), every active derivative of AANs exhibited favorable binding properties. All four derivatives showed comparable binding interactions with PTR1 according to docking and bioactivity studies. The good drug-likeness of the synthesised bioactive compounds was demonstrated by an *in silico* study of their ADME (absorption, distribution, metabolism, and excretion) characteristics.

**Fig. 7:**
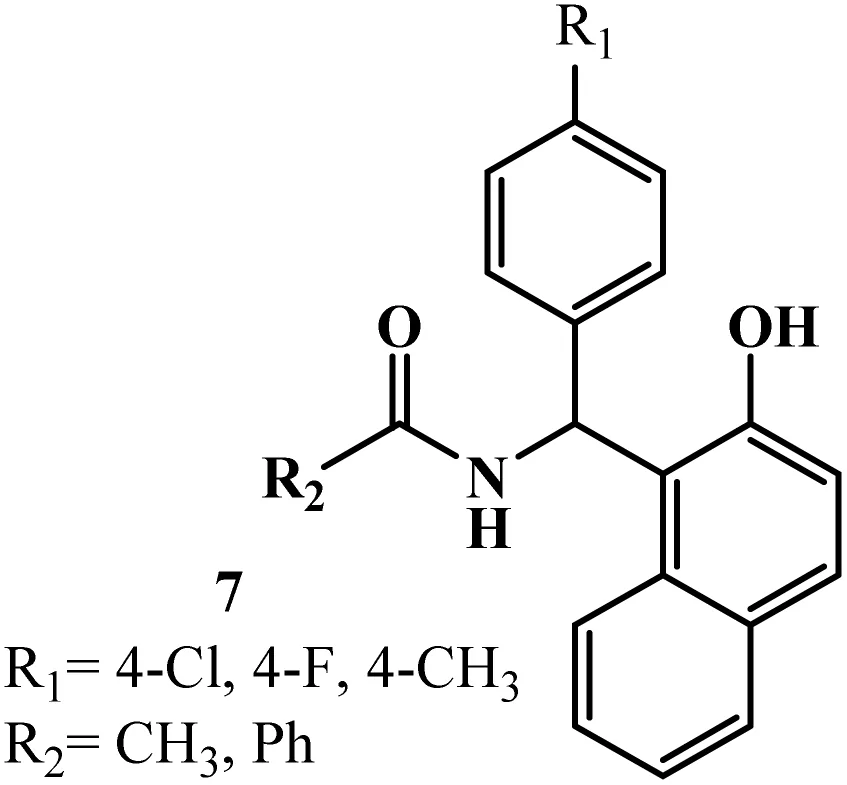
AANs with antiparasitic properties.

AAN derivatives were synthesised by Boudebbous *et al.* and tested for their cholinesterase and α-glucosidase inhibitory activities.^69^ Derivatives 8 and 9 (Fig. 8) were discovered to have up- and forthcoming features since both molecules demonstrated greater inhibition of cholinesterase activity than did the medicine galantamine. The most effective compound in the β-glucosidase experiment was compound 9, which also exhibited a greater inhibitory effect than the reference compounds quercetin and acarbose.

**Fig. 8:**
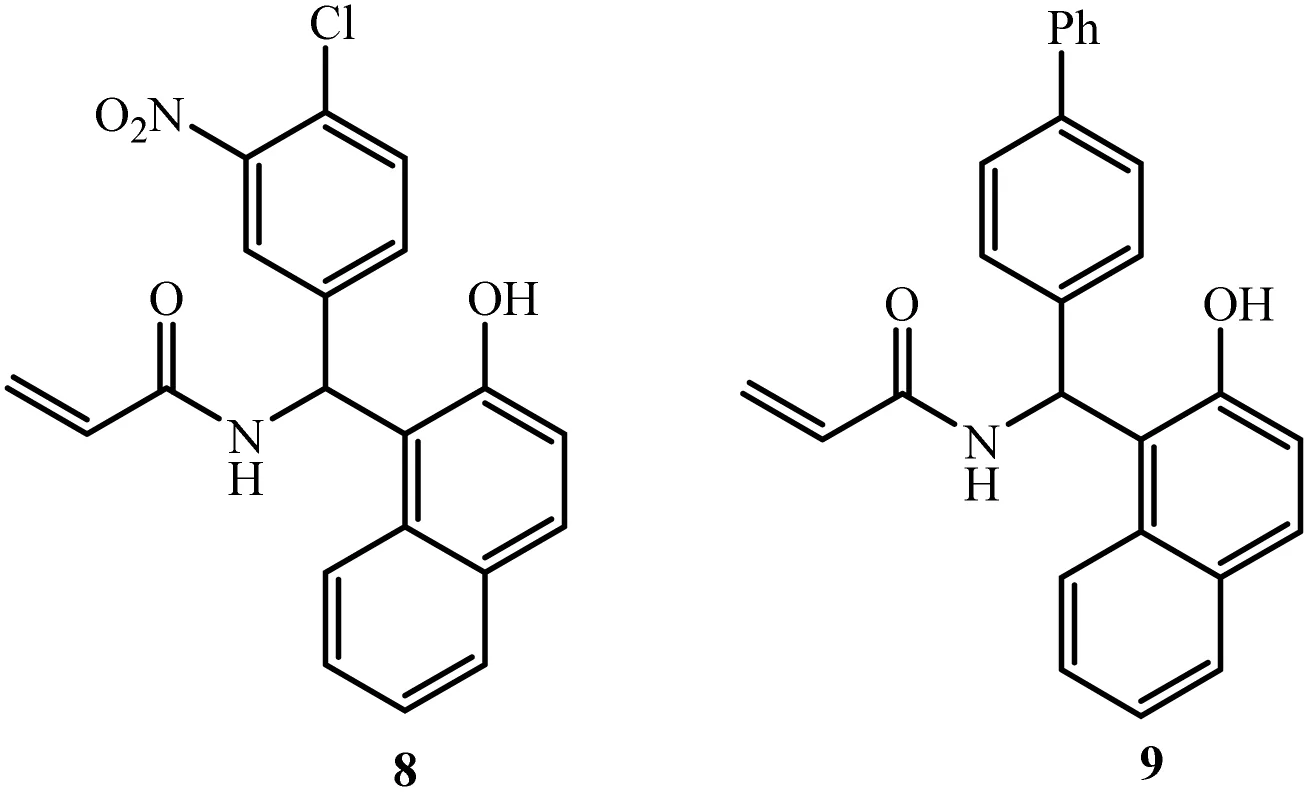
AANs act as antioxidants. Given their robust biological activity, AANs have the potential to play key roles in the pharmaceutical industry.

### Synthesis of amidoalkyl naphthols *via* a multicomponent reaction

Other methods, such as the use of catalytic transition metals, have been reported to produce compounds with comparable structures, including phenols, lawsone, 4-hydroxycoumarin, and naphthols.^76–79^ Amidoalkyl synthesis is still accomplished mainly *via* the multicomponent Mannich reaction. In organic synthesis, multicomponent reactions play a significant role in the creation of complex and varied molecules by forming bonds between carbon atoms and heteroatoms. These reactions involve the simultaneous mixing of three or more substances in a single vessel, in which they react to produce the desired end product without intermediate separation. Therefore, one-pot multicomponent synthesis adheres to the principles of green chemistry, which demands the creation of reaction conditions and new compounds that may improve chemical synthesis to meet operational simplicity, product selectivity, resource efficiency, and energy efficiency requirements. Additionally, environmental and health protections include minimizing the use of harmful solvents and reducing the amount of waste created.^80–84^

The Mannich condensation reaction involves the reaction of aldehydes with amide derivatives and 2-naphthol *via* a catalyst and is utilised as one pathway for the synthesis of AANs^85^ (Fig. 9). When carbamates are used as reaction partners, it is possible to synthesise 1-carbamatoalkyl-2-naphthols *via* the same procedure.^86–91^ However, little research has been conducted on the preparation of 1-carboxyalkyl-2-naphthols in the last three years.^92,93^ The three-component Mannich condensation of amides with 2-naphthol and aldehydes yields the primary production of highly reactive *ortho*-quinone methides, as per the generally accepted reaction mechanism. These intermediates generate 1-amidoalkyl-2-naphthol *via* nucleophilic addition to an amide (Fig. 9).^94–96^ This reaction has been effectively promoted by various catalytic systems, according to reports. These catalysts can be generally classified into two categories: (1) homogeneous (Brønsted acids and ionic liquids) and (2) heterogeneous (nanomaterials, *etc.*).

**Fig. 9:**
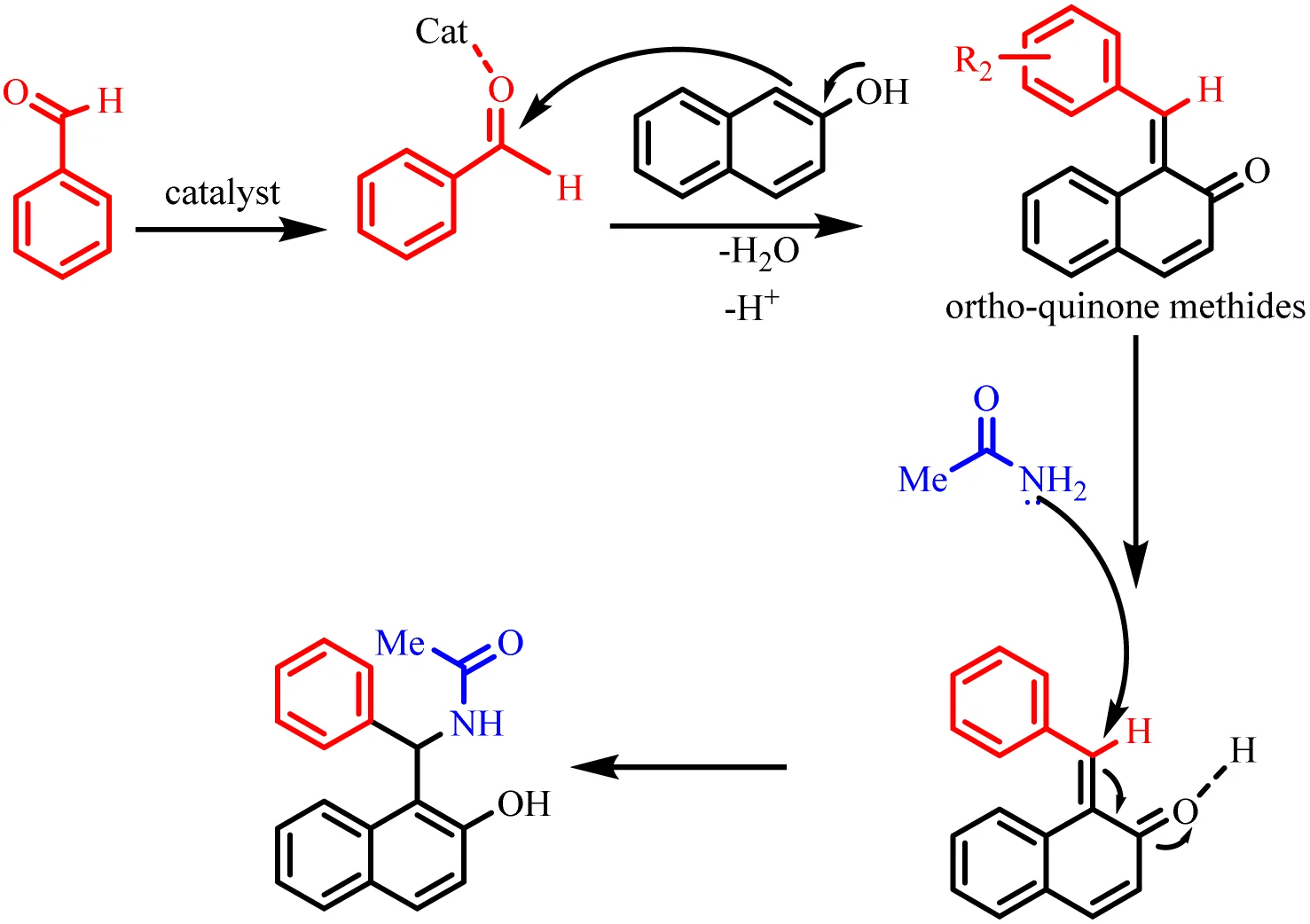
Mechanism of the preparation of AANs *via* the multicomponent Mannich method.

This reaction is catalyzed by either Brønsted acids, Lewis acids, or solid acid heterogeneous catalysts under thermal or ultrasonic conditions.

Step 1: formation of the imine/iminium ion (activation of aldehyde): the catalyst (Brønsted or Lewis acid) activates the aromatic aldehyde by coordinating to the carbonyl oxygen, increasing the electrophilicity of the carbonyl carbon. The amide (or urea) then attacks the activated aldehyde to form a hemiaminal intermediate, which upon dehydration (loss of H_2_O) generates an iminium ion (or protonated Schiff base). Alternatively, the aldehyde can condense directly with the amide to form an imine intermediate. The role of the catalyst here is to facilitate the nucleophilic attack of the amide on the aldehyde and to promote the elimination of water to form the electrophilic iminium species.

Step 2: formation of the *ortho*-quinone methide (*o*-QM) intermediate: the 2-naphthol undergoes tautomerization to its enol form (or keto form), which is favored in the presence of acid. The enol form of 2-naphthol then attacks the electrophilic iminium ion in a Friedel–Crafts-type alkylation at the *ortho*-position (C-1 of the naphthol ring), leading to a benzylic alcohol-type adduct. This adduct undergoes dehydration (loss of H_2_O) to generate the highly reactive *ortho*-quinone methide (*o*-QM) intermediate. This is the key reactive species in the mechanism. The role of the catalyst here is to promote the enolization of 2-naphthol and assist in the dehydration step by stabilizing the leaving water molecule (through hydrogen bonding or coordination), thereby facilitating the formation of the *o*-QM intermediate.

Step 3: nucleophilic addition of the amide (final product formation): the *o*-QM intermediate is highly electrophilic at the benzylic carbon. A second equivalent of the amide (or urea) acts as a nucleophile and attacks the *o*-QM at the benzylic position. Proton transfer and rearomatization of the naphthalene ring occur, yielding the final 1-amidoalkyl-2-naphthol (AAN) product. The role of the catalyst here: the acid catalyst activates the *o*-QM for nucleophilic attack and facilitates the final proton transfer steps, ensuring rearomatization and product stability (Fig. 10).

**Fig. 10:**
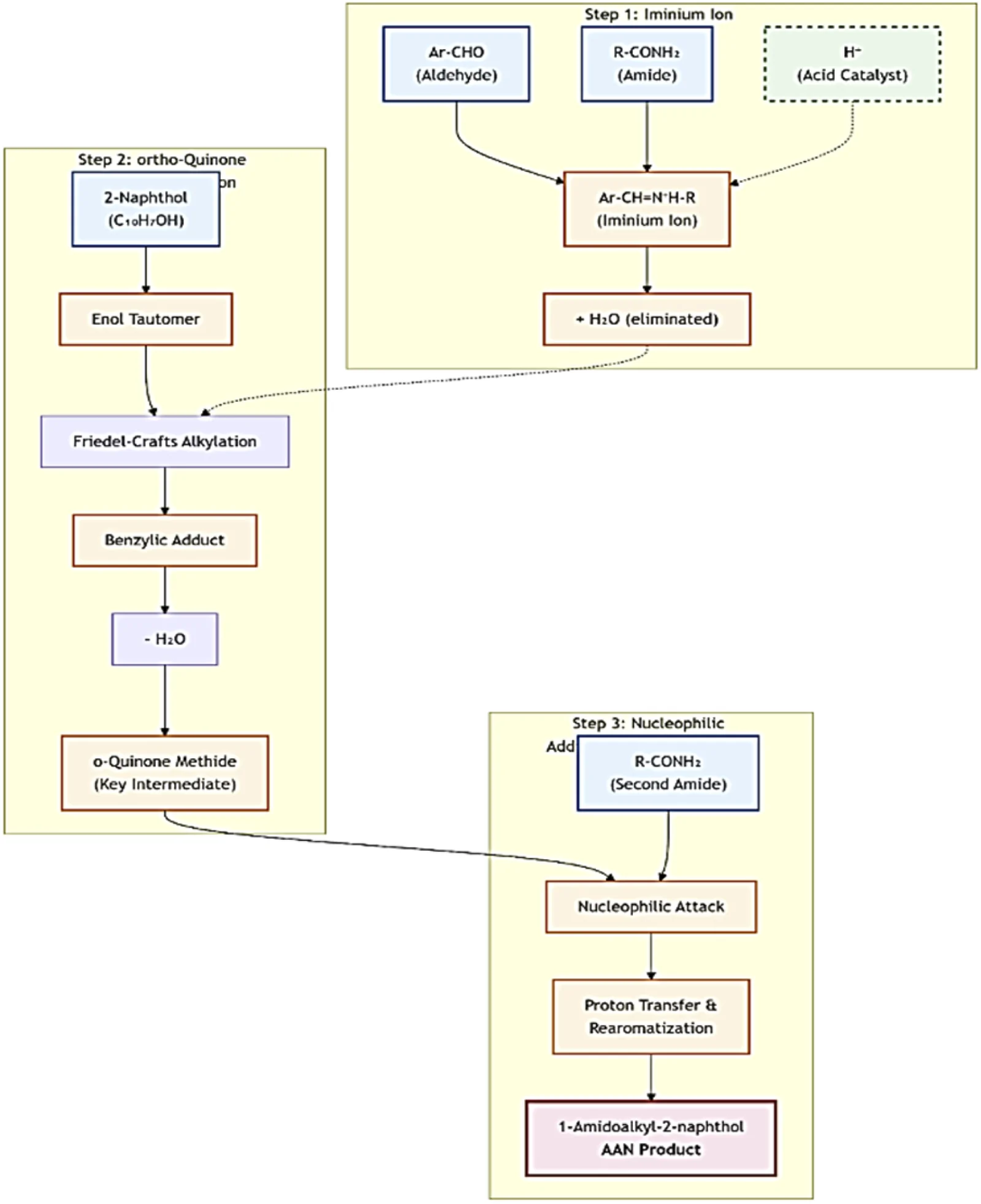
General reaction mechanism, brief mechanistic discussion and the role of catalysts in multicomponent reactions.

### Homogeneous catalysis

When a catalyst is in the same phase as the reactants, it is said to be homogeneous. Their ability to activate a wide range of functional groups makes them useful in chemical synthesis. One of the main benefits of homogeneous catalysts is that they are often stable in the presence of both oxygen and water. Because they are metal-free, they also serve as valuable substitutes for metal catalysts in the medical industry, where eliminating hazardous metal impurities from the finished product completely can be challenging. Examples of homogeneous catalysts utilised in studies to create AANs include ionic liquids, Brønsted acids such as sulfamic acid, and molecular iodine.^52^ Das *et al.*^97^ developed an operationally easy method. A mixture of molecular iodine (5 mol%, catalyst), amide or urea (1.3 mmol), aldehyde (1 mmol), and 2-naphthol (1 mmol) in dichloroethane (10 mL) was used. This reaction was carried out at room temperature to synthesize AANs in high yield (Scheme 1).

**Scheme 1:**
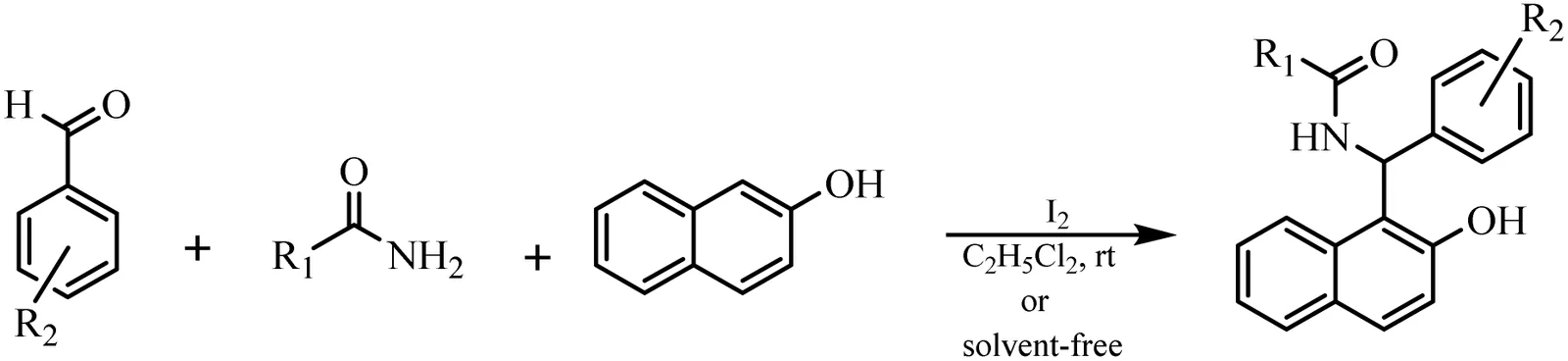
Molecular iodine-mediated preparation of AANs *via* multiple components.

Short reaction times and good yield of AANs are achieved by a three-component one-pot condensation of urea/amides, aldehydes, and 2-naphthol, catalysed by sulfamic acid (2.5 mmol) under ambient conditions and ultrasonic irradiation (Scheme 2).^98^

**Scheme 2:**
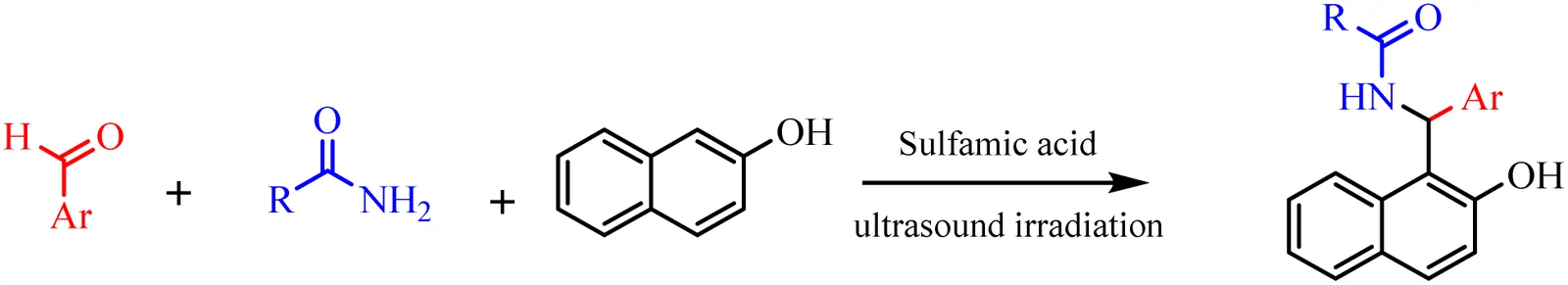
Preparation of AANs using sulfamic acid.

One study was conducted under solvent-free conditions through the multicomponent condensation of (hetero)aromatic aldehydes, 2-naphthol, and various combinations of thiourea/urea/2-aminothiazole/acetamide/4-nitrothiazol-2-amine/2-aminopyridine. The significant aspects of this approach include the absence of column chromatography, excellent yields, minimal cost, and the use of a (triethylammonium acetate, 20 mol%) ionic liquid as a clean, green, and reusable catalyst (Scheme 3).^64^

**Scheme 3:**
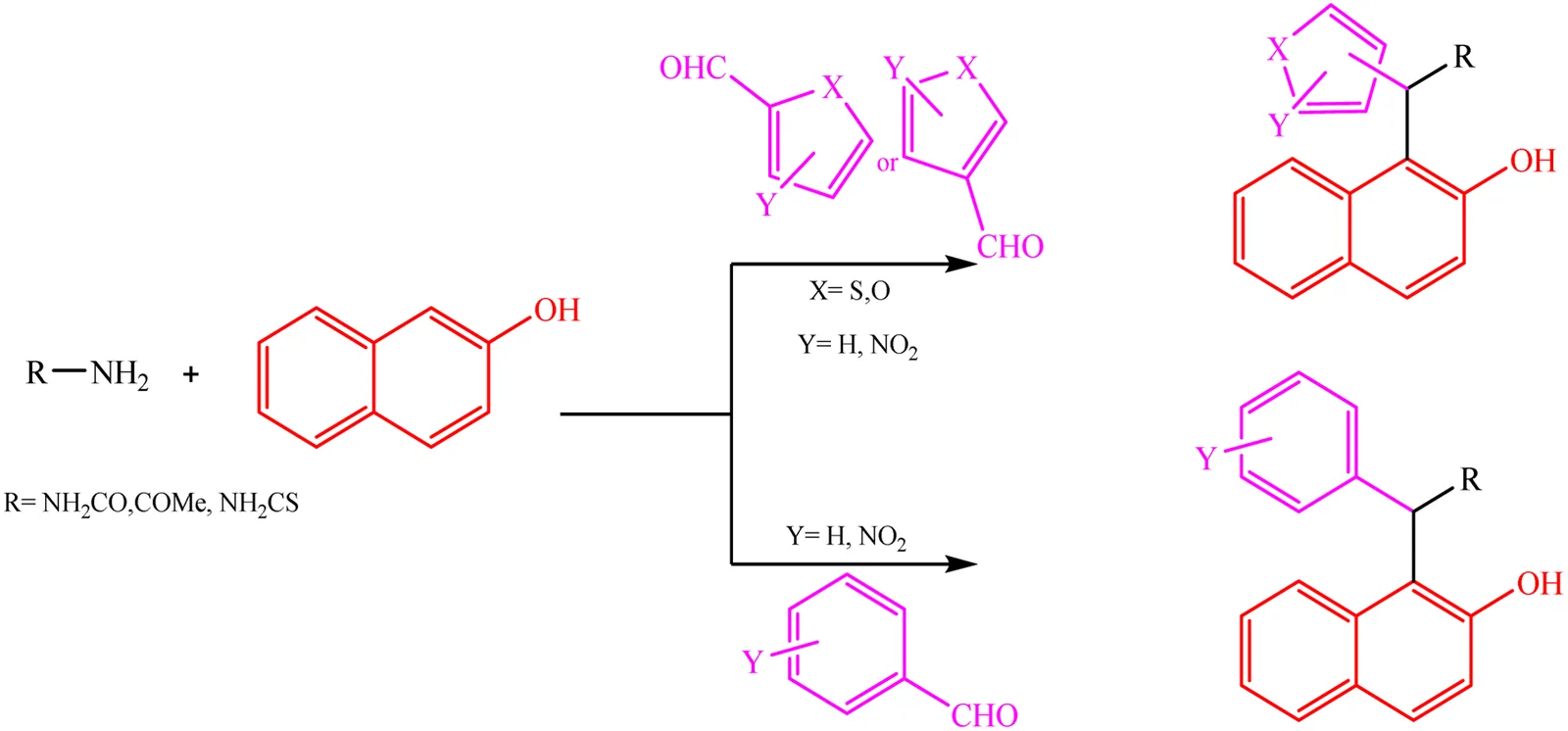
Preparation of AANs *via* triethylammonium acetate.

High yields of AANs were synthesised by Jiang *et al.*^99^ A mixture of 2-naphthol, aldehyde, urea, or amide was condensed using molybdophosphoric acid (heteropolyacid) (0.12 g) as a catalyst (Scheme 4).

**Scheme 4:**
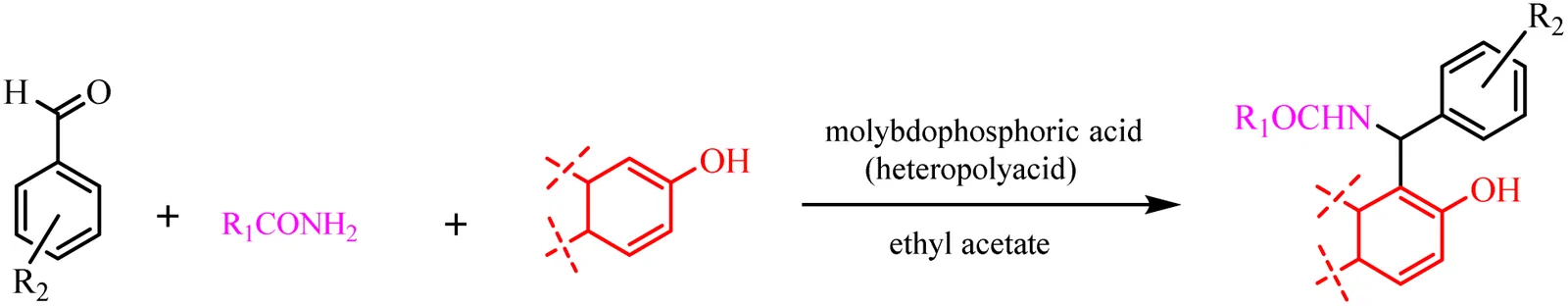
Preparation of AANs catalyzed by molybdophosphoric acid.

A simple, cost-effective, and efficient technique for the preparation of β-thiocarbamidoalkyl-β-naphthols using *p*-toluenesulfonic acid (*p*-TSA) as a catalyst at room temperature has been reported. N-Phenylthiourea or thiourea, aromatic aldehydes, and β-naphthol have been used to produce a range of thiocarbamidoalkyl naphthols in elevated yields. This technique involves a short reaction time and is a simple experimental technique (Scheme 5).^100^

**Scheme 5:**
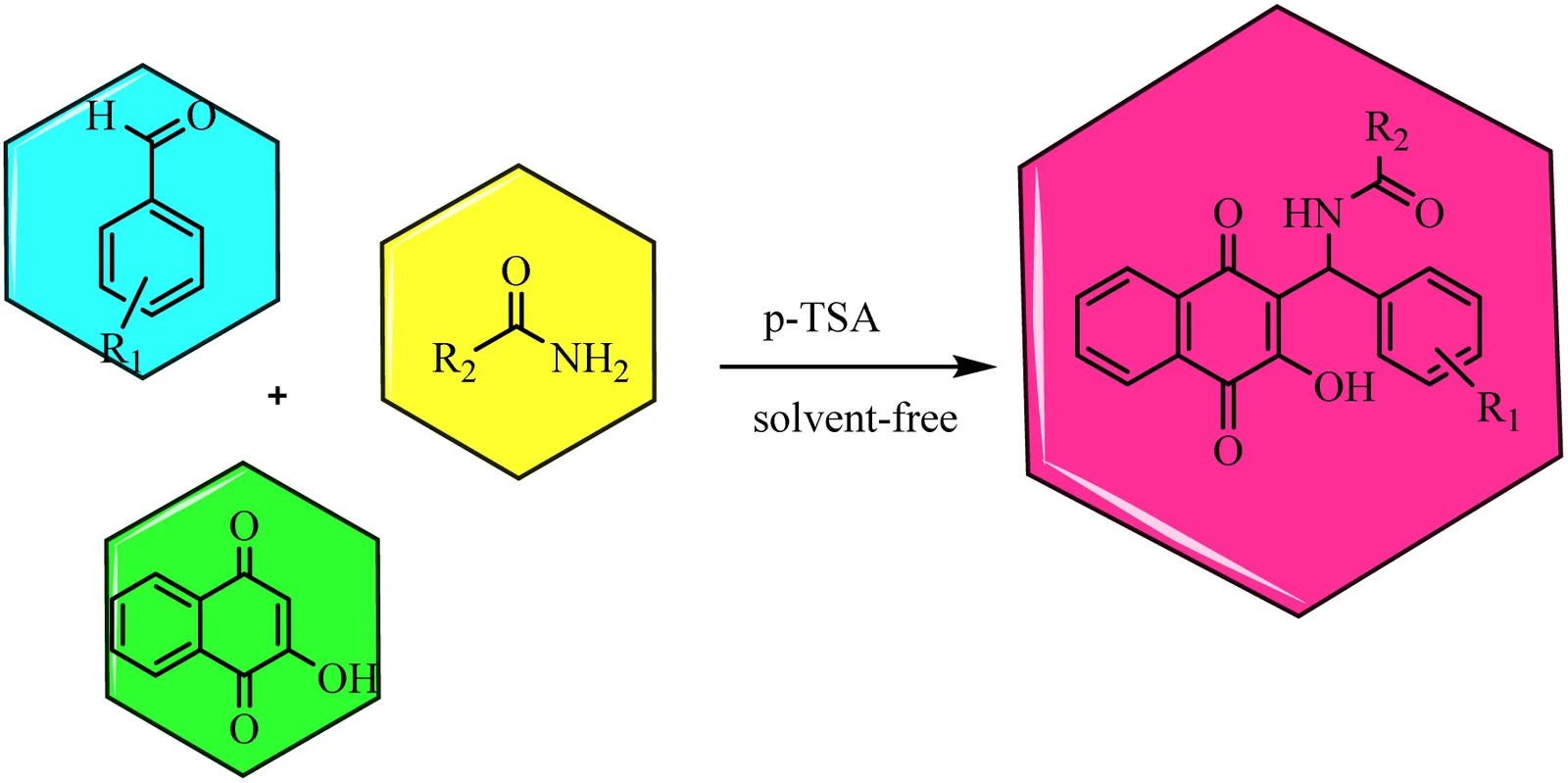
MCR-based AAN synthesis *via p*-TSA.

Under solvent-free conditions, 2-naphthol, benzamide or acetamide, and aromatic aldehydes were used to prepare the corresponding AANs. The optimum catalyst to facilitate the reaction was discovered to be adipic acid (Scheme 6).^101^

**Scheme 6:**
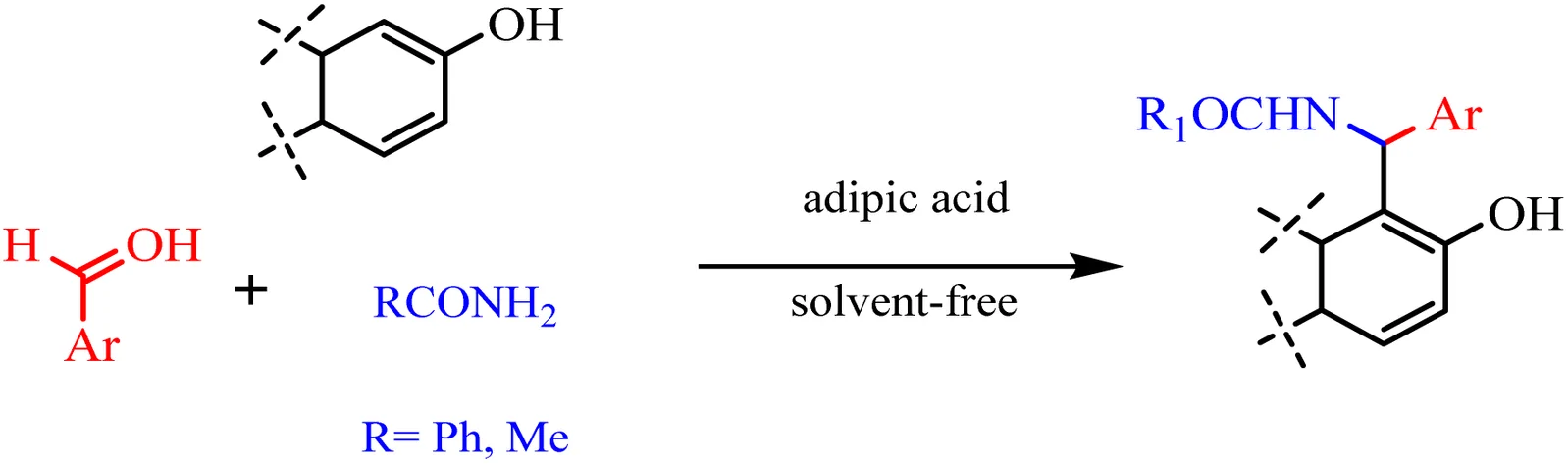
Adipic acid as a catalyst for the synthesis of AANs.

Under solvent-free conditions, a mixture of aldehyde, 4-hydroxycoumarin, benzaldehyde, and acetamide was reacted *via* (*p*-TSA, 30 mol%) catalysis, resulting in the formation of 3-(α-amidobenzyl)-4-hydroxycoumarin in high yield. This solvent-free synthesis represents a more sustainable and eco-friendly approach than traditional solvent-based methods (Scheme 7).^102^

**Scheme 7:**
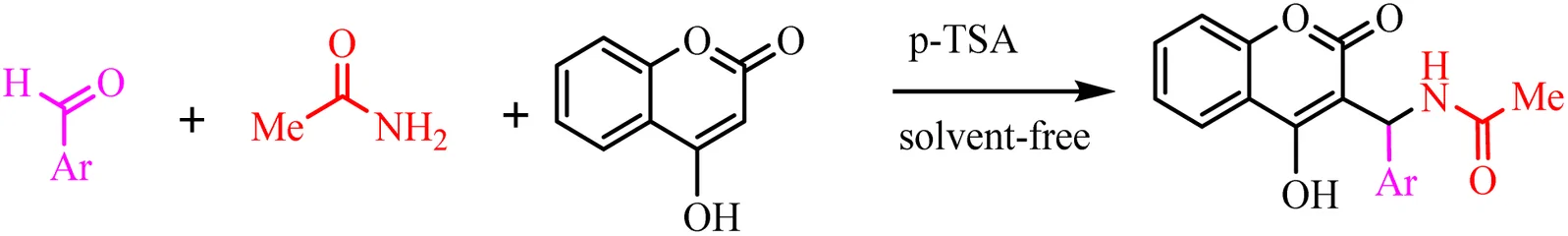
*p*-TSA as a catalyst for the preparation of AANs.

A newly developed, efficient synthesis of AAN derivatives *via* a solvent-free protocol employing phenylboronic acid as a catalyst (15 mol%) was reported. This methodology uses phenylboronic acid as a catalyst to facilitate the reaction of AAN precursors under solventless conditions, giving high yields of the desired products without the need for organic solvents. Compared with conventional methods, this approach enhances the method's sustainability and eco-friendly characteristics. Furthermore, the inhibitory potential of the selected compounds against acetylcholinesterase (AChE) and α-glucosidase was evaluated (Scheme 8).^69^

**Scheme 8:**
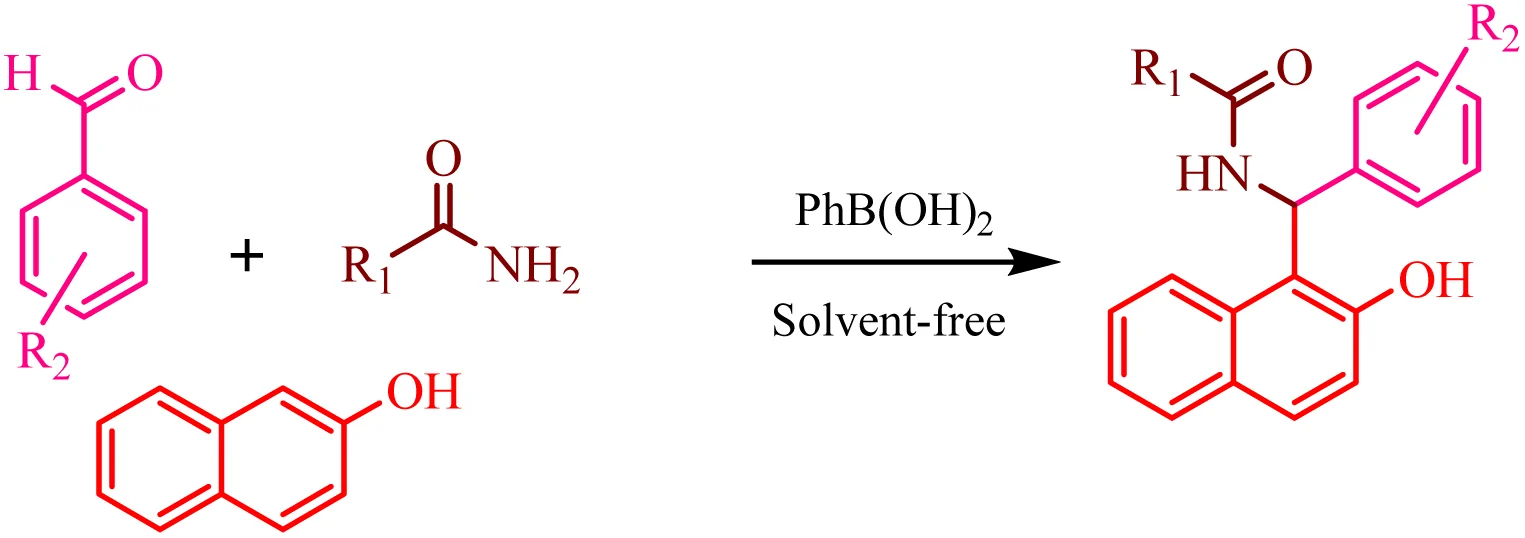
Preparation of AANs using the phenylboronic acid as a catalyst.

Biologically significant amidoalkyl naphthoquinones were synthesized by Zhaleh *et al.*^37^ in a new and environmentally friendly approach. This method involves a one-pot multicomponent reaction of aromatic aldehydes, lawsone (2-hydroxy-1,4-naphthoquinone), and benzamide or acetamide at 80 °C under solvent-free conditions in the presence of *p*-TSA (15 mol%). This straightforward and efficient protocol is anticipated to be effective for the combinatorial synthesis of bioactive compounds, as amidoalkyl naphthoquinone derivatives are a crucial class of naturally occurring compounds. The significance of this discovery lies in the fact that these compounds possess a wide range of biological activities, including anticancer, antimicrobial, and antioxidant properties (Scheme 9).

**Scheme 9:**
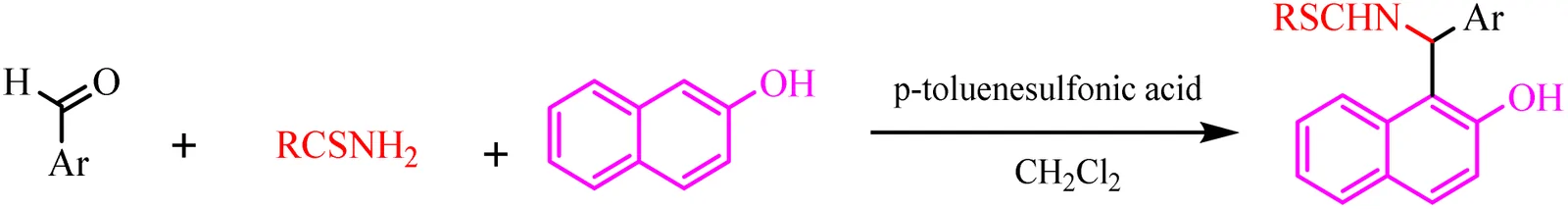
Preparation of AANs *via p*-TSA.

Amidoalkyl compounds are essential for the production of potent drugs and crucial biological substances. In light of this, a new approach to synthesising AANs and amidoalkyl naphthoquinones using ascorbic acid (8.5 mol%) or vitamin C as a natural, water-soluble catalyst under solvent-free conditions has been developed. This method offers several advantages, including a short reaction time, high product yield, easy workup, and low catalyst cost (Scheme 10).^103^

**Scheme 10:**
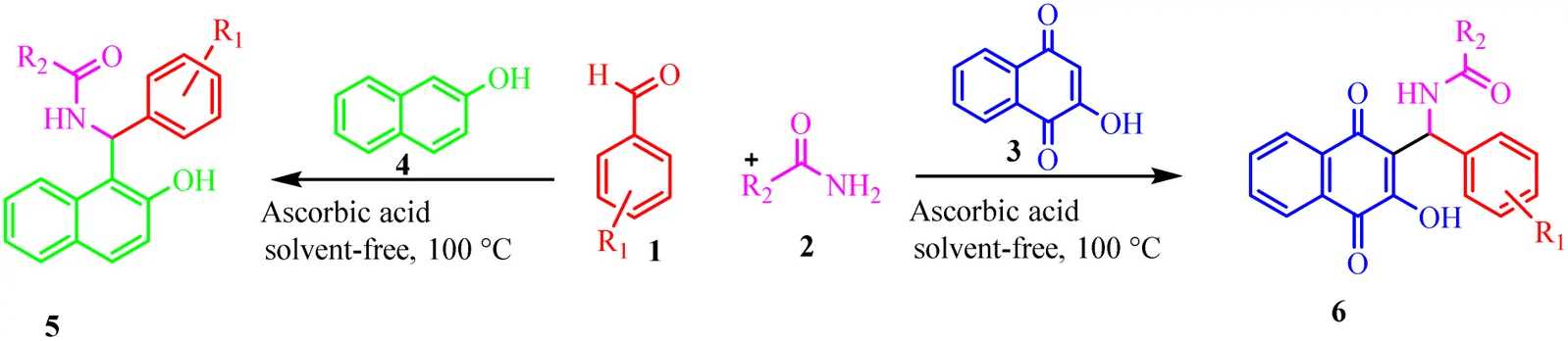
Ascorbic acid-catalyzed AAN synthesis.

### Ionic liquids as catalysts and solvents

Owing to their unique chemical and physical properties, ionic liquids are often utilised as a green reaction environment; nevertheless, they have now surpassed this limitation and have proven their importance in reaction control.^104^ Ionic liquids are advantageous as catalysts for a variety of chemical reactions because of their biodegradability, nontoxicity, and ease of manufacture.^105^

A more straightforward and efficient method for synthesizing AANs through a one-pot, three-component reaction of aromatic aldehydes, β-naphthol, and amides in ionic liquids, using ytterbium triflate as a mild Lewis acid catalyst, has been developed. This process offers several benefits, including high yields, eco-friendliness, affordability, and noncorrosive conditions. Additionally, the catalyst can be easily recycled (Scheme 11).^106^

**Scheme 11:**
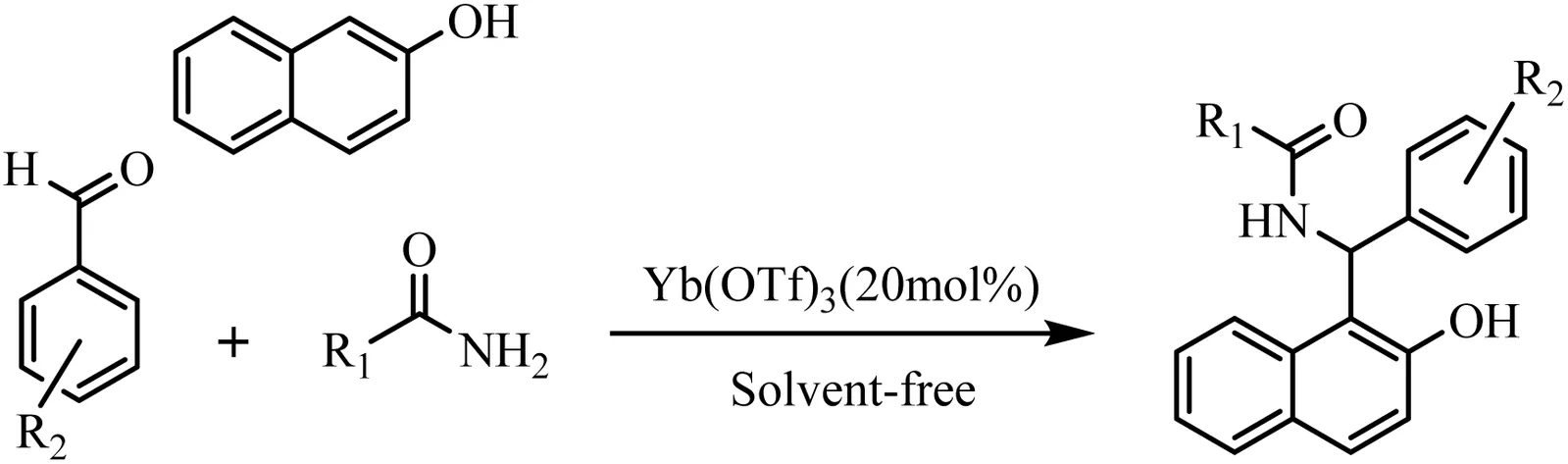
Preparation of AANs using Yb(OTF)_3._

A new, mild, and productive approach has been devised for synthesizing AANs by combining amides or urea, aldehydes, and 2-naphthol in the presence of a small amount of a Brønsted acidic ionic liquid ([TEBSA][HSO_4_] 5 mol%) under solvent-free and thermal conditions. This method offers several advantages, including high yields, short reaction times, simple work-up procedures, and the ability to reuse the catalyst (Scheme 12).^55^

**Scheme 12:**
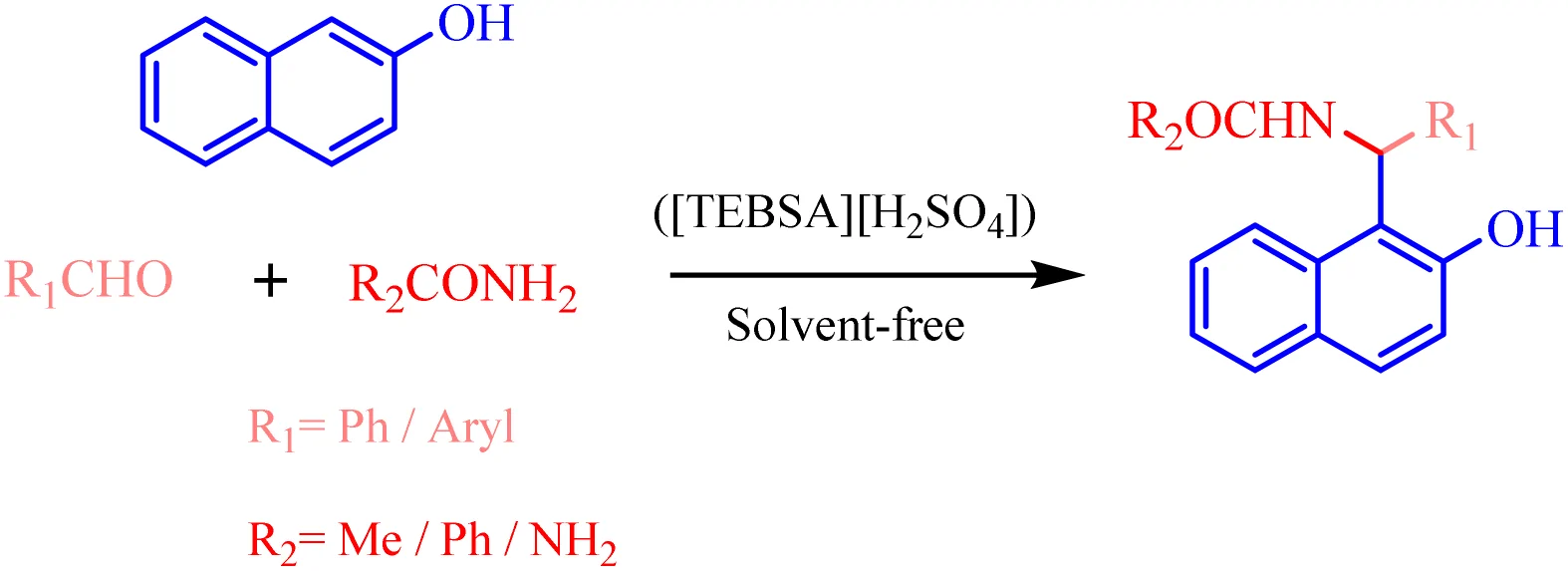
[TEBSA][HSO_4_] as a catalyst for the preparation of AANs.

Khazdooz *et al.*^107^ developed a novel and efficient approach for the synthesis of AANs *via* a one-pot, three-component condensation reaction between aromatic aldehydes, amides, or urea and 2-naphthol in the presence of a catalytic amount of the Brønsted acidic ionic liquid [Himin][HSO_4_] under solvent-free conditions. This method offers several advantages, including the use of nontoxic, affordable materials, a straightforward, clean workup, short reaction times, and high product yields. This procedure is both eco-friendly and economical (Scheme 13).

**Scheme 13:**
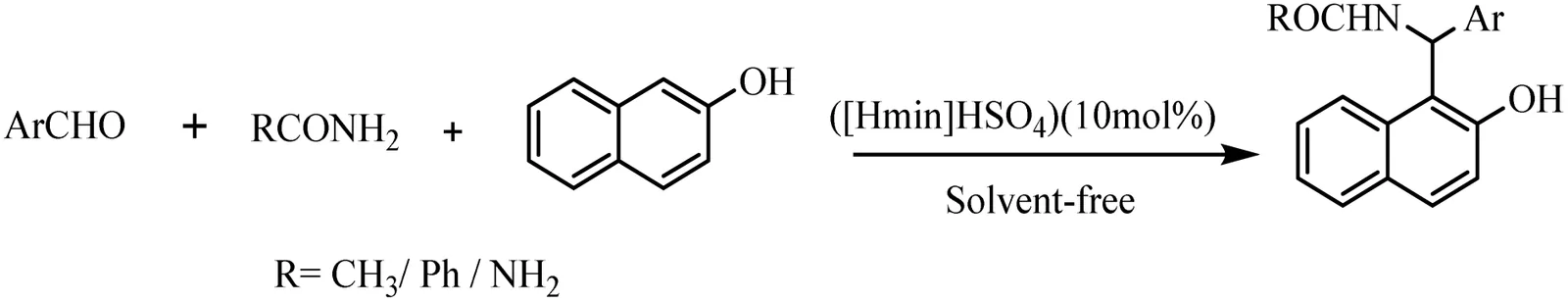
Preparation of AANs using [Himin][HSO_4_].

The synthesis of AANs was accomplished through a one-pot multicomponent condensation reaction between aromatic aldehydes, β-naphthols, and amide derivatives catalysed by sulfonic acid-functionalized imidazolium salts under solvent-free conditions. The efficient catalysts employed in this study include {[Msim]Cl}, {[Dsim]Cl}, and {[Msim]AlCl_4_}. The use of these catalysts results in excellent yields of the desired products and remarkably short reaction times (Scheme 14).^45^

**Scheme 14:**
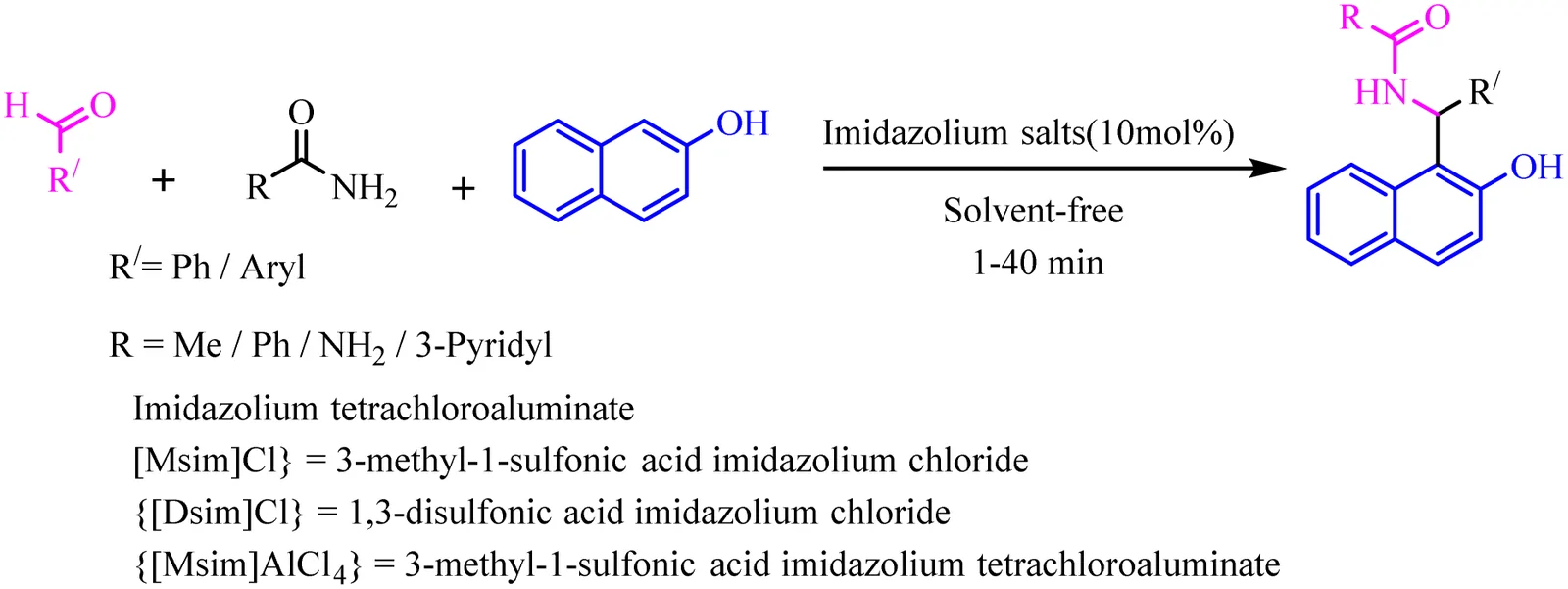
Imidazolium salts as catalysts for the preparation of AANs.

An effective new method was developed for synthesizing AANs through a single-pot, three-component condensation reaction involving 2-naphthol, amides, and aromatic aldehydes in the presence of a dicationic acidic ionic liquid based on polyethene glycol (PEG) as a catalyst under solvent-free conditions. This method has several noteworthy features, including high yields, short reaction times, simple work-up procedures, and reusability of the catalyst (Scheme 15).^56^

**Scheme 15:**
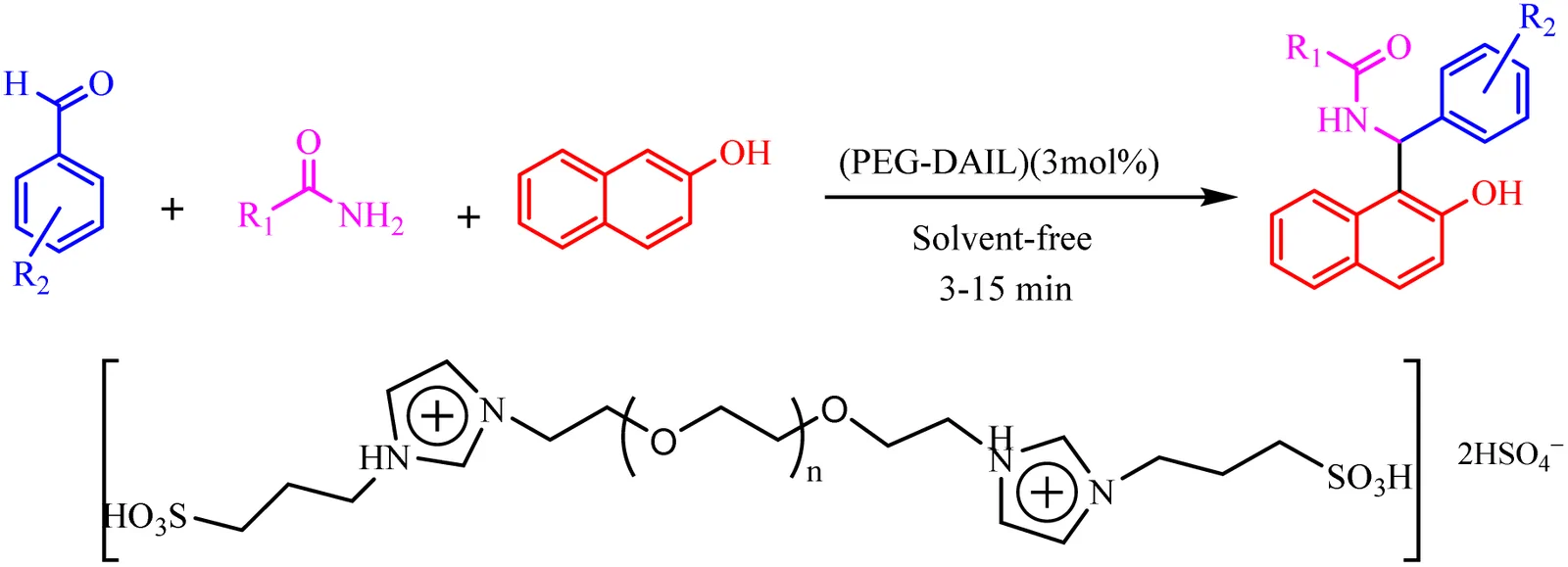
Preparation of AANs using PEG.

The high catalytic activity of [Tmim] [HSO_4_]_4_ as a tetradentate acidic catalyst was demonstrated by Savari and Heidarizadeh. Under solvent-free conditions, at 90 °C, only 1.25 mol% of the catalyst was required for the production of AANs. This catalyst possesses several intriguing characteristics, including the ability to form hydrogen bonds, high catalytic efficiency, distinct heterogeneous properties, and applicability in solvent-free environments (Scheme 16).^108^

**Scheme 16:**
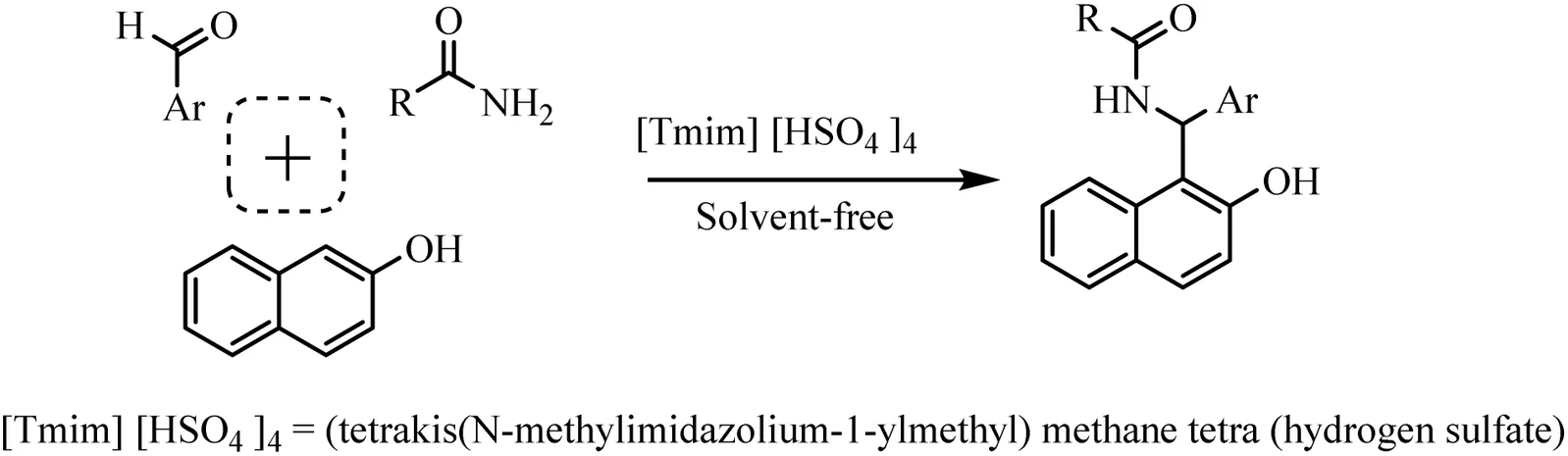
Multicomponent synthesis of AANs using [Tmim] [HSO_4_]_4_.

Triethylammonium hydrogen sulfate ([Et_3_NH] [HSO_4_]) is an eco-friendly, recyclable catalyst that does not require column chromatography and offers high efficiency, good yield, low cost, and simple processing; thus, it is an excellent choice. A multicomponent reaction between aromatic aldehydes, urea or acetamide, and 2-naphthol produces AAN compounds. This reaction occurred at 70 °C with a catalyst amount of 20 mol% (Scheme 17).^109^

**Scheme 17:**
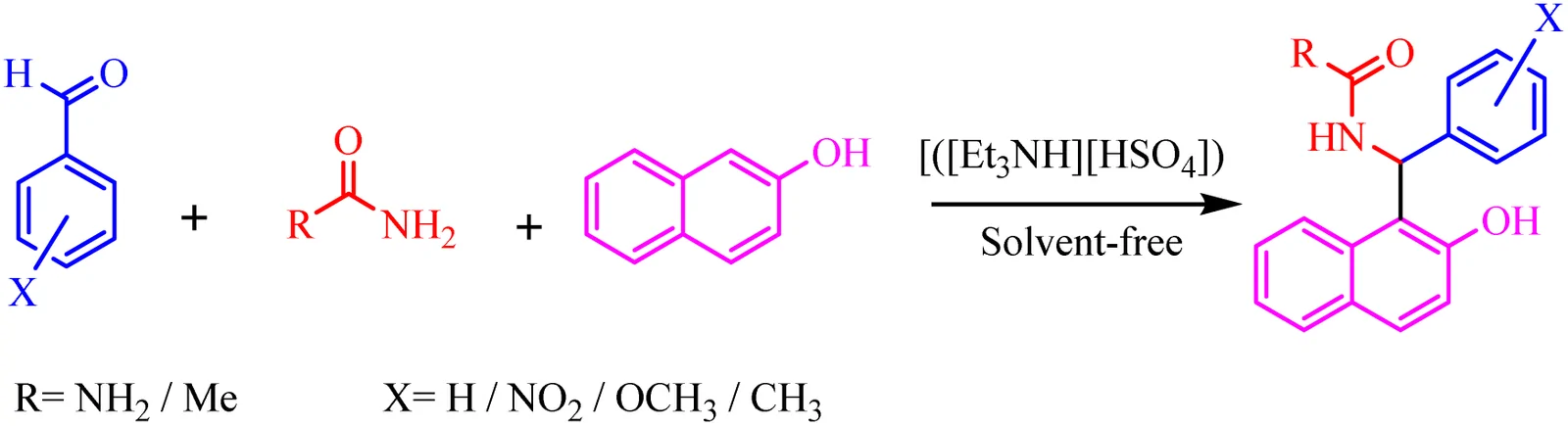
[Et_3_NH] [HSO_4_]-catalyzed AANs synthesis.

[TMEDSA][Cl]_2_ was employed as an efficient catalyst to facilitate organic reactions under environmentally friendly, solvent-free conditions: (i) the preparation of α-carbamatoalkyl-2-naphthols by reacting arylaldehydes, 2-naphthol, and alkyl carbamates, and (ii) the production of AANs by combining arylaldehydes, 2-naphthol, and amides (Scheme 18).^92^

**Scheme 18:**
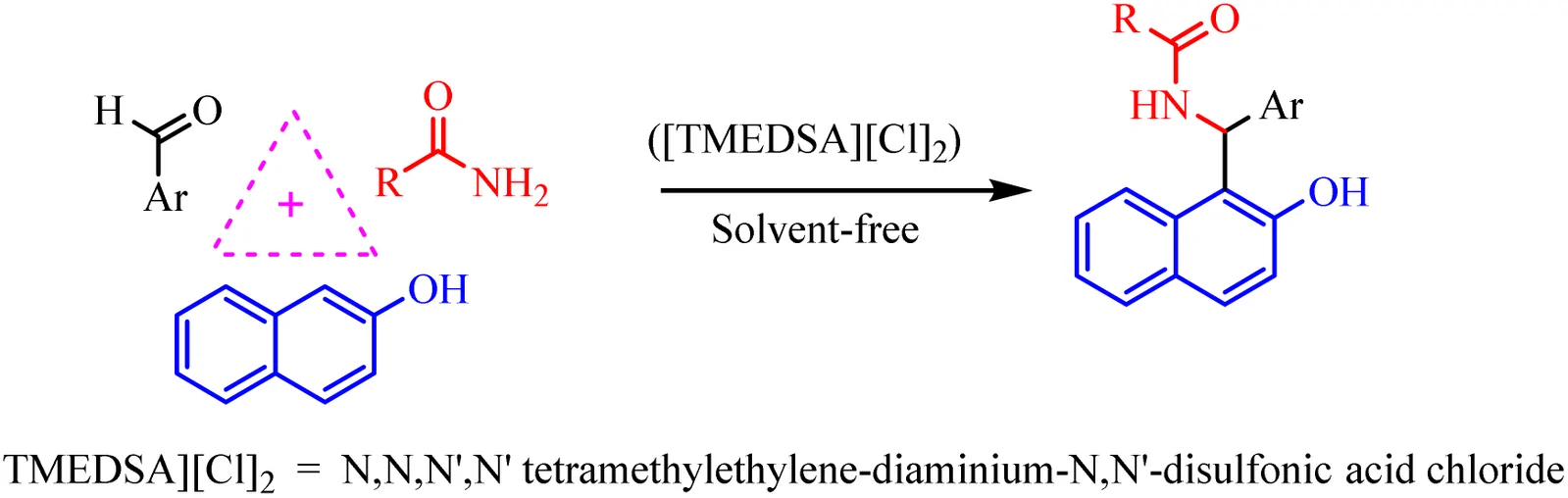
Preparation of AANs using [TMEDSA][Cl]_2_.

In a study, a new type of magnetic phosphonium ionic liquid (MPIL) was created *via* a two-step process. This MPIL has dual acidic properties and shows excellent catalytic activity in the preparation of AAN and 2′-aminobenzothiazolomethylnaphthol compounds. The combination of magnetic properties and Brønsted and Lewis acids in a single compound is a significant advantage of the prepared MPIL. This research highlights the potential for designing and applying multifunctional magnetic ionic liquids for various purposes (Scheme 19).^110^

**Scheme 19:**
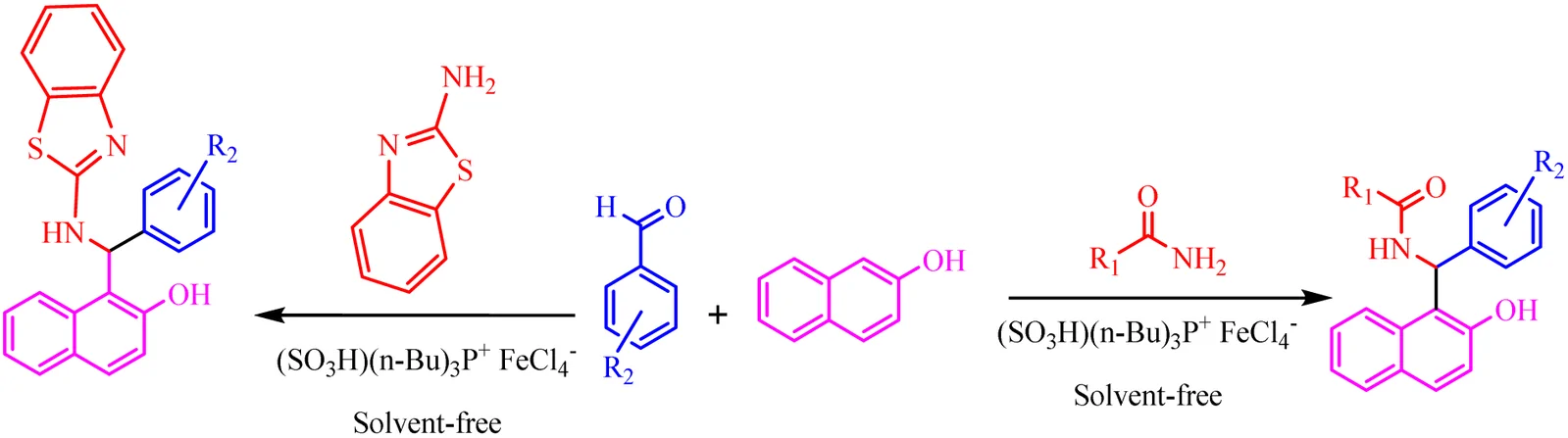
Magnetic phosphonium ionic liquids for the synthesis of AANs.

Nguyen *et al.* synthesized AANs^111^*via* a multicomponent reaction involving an aromatic aldehyde, 2-naphthol and 2-chloroacetamide/benzamide in combination with [cholineCl][ZnCl_2_] as a catalyst (20 mol%). This method provides several benefits, including high yields, reusability of the catalyst, and environmental friendliness (Scheme 20). As summarized in Table 2, ionic liquid catalysts combine the advantages of homogeneous and heterogeneous systems, offering high thermal stability, negligible vapor pressure, and facile recyclability.

**Scheme 20:**
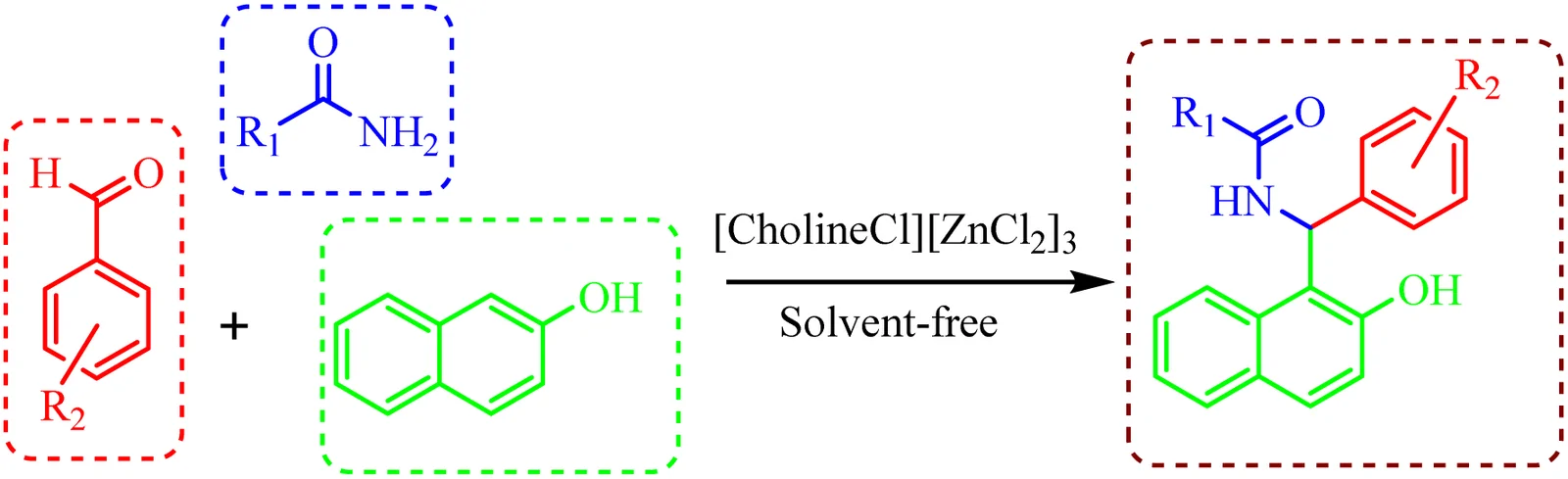
Preparation of AANs with [cholineCl][ZnCl_2_]_3_.

### Heterogeneous catalysis

The current challenges can be summarized as follows: homogeneous catalysts are effective but not recyclable. While homogeneous catalysts are often highly effective due to their excellent selectivity and activity (since the active site is well-defined and fully accessible to the reactants), a significant drawback is their limited recyclability, which directly affects their score in quantitative green metrics. While homogeneous catalysts such as molecular iodine or sulfamic acid offer high yields (up to 94%) and shorter reaction times, they consistently fail the recyclability criteria, as shown by a comparison of Table 1 (homogeneous) and Table 2 (ionic liquids/heterogeneous). On the other hand, heterogeneous systems, especially magnetic nanocatalysts such as Fe_3_O_4_@RHA@MEL-SO_3_H (Table 3), do not compromise catalytic efficiency in pursuit of sustainability. These methods dramatically reduce the process's E-factor while maintaining yields above 90% and making recovery simple *via* magnetic separation.

**Table 1:** Homogeneous catalysis^a^

Entry	Catalyst, mol%	Reusability	Medium	*T*, °C	Time	Yield, %	Ref.
1	Molecular iodine (5)	NO	S-F or C_2_H_5_Cl_2_	125 or r.t	4–9 h or 10–26 h	20–90 or 35–93	97
2	Sulfamic acid (2.5 mmol)	NO	US	30	15–120 min	55–94	98
3	Triethylammonium acetate (20)	NO	S-F	90	40–90 min	60–91	64
4	Molybdophosphoric acid (0.12 g)	NO	Ethyl acetate	65	3–4 h	86–97	99
5	*p*-Toluenesulfonic (10)	NO	CH_2_Cl_2_	r.t	8–24 h	58–93	100
6	Adipic acid (10)	NO	S-F	120	9–76 min	83–96	101
7	*p*-Toluenesulfonic (30)	NO	S-F	115	4.5–5.5 h	80–90	102
8	Phenylboronic acid (15)	NO	S-F	120	1–7 h	58–91	69
9	PTSA (15)	NO	S-F	80	24–60 min	68–88	37
10	Ascorbic acid (8.5)	NO	S-F	100	4–12 min	75–96	103

a S-F: Solvent free, US: ultrasound irradiation.

**Table 2:** Ionic liquids as solvents and catalysts

Entry	Catalyst mol%	Reusability	Medium	*T*, °C	Time	Yield, %	Ref.
1	Yb(OTf)_3_ (20)	No	Solvent-free	80	4–12 h	35–92	106
2	([TEBSA][H_2_SO_4_]) (5)	No	Solvent-free	120	10–45	73–91	55
3	([Hmin]HSO_4_) (10)	Not reported	Solvent-free	115	15–30	78–93	107
4	Imidazolium salts (10)	No	Solvent-free	120	1–40	81–96	45
5	(PEG-DAIL) (3)	No	Solvent-free	80	3–15	81–98	56
6	[Tmim] [HSO_4_ ]_4_ (1.25)	No	Solvent-free	90	3–15 min	90–96	108
7	([Et_3_NH][HSO_4_]) (20)	Yes (3)	Solvent-free	70	2–25 min	65–85	109
8	{[TMEDSA][Cl]_2_} (7.5)	No	Solvent-free	70	15–25 min	92–95	93
9	(SO_3_H) (*n*-Bu)_3_P^+^ FeCl_4_^−^ (1)	No	Solvent-free	45	5–30 min	75–94	110
10	[CholineCl][ZnCl_2_]_3_ (20)	Yes (3)	Solvent-free	60	40–80 min	45–95	111

**Table 3:** Heterogeneous catalysis

Entry	Catalyst, mol%	Reusability	Medium	*T*, °C	Time	Yield, %	Ref.
1	Fe(HSO_4_)_3_ (5)	Yes (5)	CH_3_CN, reflux	85	20 h	47–74	48
			S-F M.W.	85	25–80 min	74–97	
				450 W	5–14 min	84–97	
2	NKC-9 (0.17 g)	Yes (4)	CHCl_3_, Reflux	85	3–10 h	82–96	44
3	(PBS) (0.04 g)	Yes (5)	S-F	120	4–9 min	85–95	58
4	AgI (0.3 g)	Yes (6)	S-F	110	3–20 min	80–95	59
5	OPec-Mel-SA (7.36)	Yes (5)	S-F	90	20–60 min	85–97	29
6	(Fe_3_O_4_@RHA@MEL-SO_3_H (0.011 g)	Yes (9)	S-F	118	3–42 min	85–92	30
7	Fe_3_O_4_@SiO_2_/APTES-Schiff bases of Cu (40 mg)	Yes (6)	Ultrasonic conditions	25	25–30 min	88–96	31
8	KCC-1/ECH-Meg/Cd(ii) (0.025 g)	Yes (5)	S-F	100	2–10	85–97	113
9	CuFe_2_O_4_/KCC-1/PMA (0.03 g)	Yes (5)	S-F	80	2–13	82–95	114
10	M-MnO_2_ NW/KCC-1/PMA (0.008 g)	Yes (5)	S-F	80	2–13	82–95	115

High effectiveness (activity and selectivity): in homogeneous catalysis, the catalyst is fully dissolved in the reaction mixture, creating a single phase. This maximized contact between the catalyst and reactants leads to very high reaction rates and often results in superior control over the stereochemistry or regioselectivity (high selectivity) of the product.

Poor recyclability (Green Metric Issue): because the catalyst is dissolved in the same phase as the product, separating the product from the catalyst is often difficult, complex, and energy-intensive (*i.e.*, high energy input).

Separation costs: the separation process typically involves chemical methods (such as precipitation, solvent extraction, or chromatography), which can lead to significant catalyst loss, reduced overall atom economy due to the use of extra solvents/reagents, and high purification costs.

Catalyst loss: high catalyst loss after one reaction cycle makes the system unsuitable for industrial application and results in a very poor recyclability score, classifying it as “nonreusable” or “single-use”.

The solution (the heterogeneous alternative): this is why the current trend, especially in green chemistry (in the context of this review on Betti-based AANs), favours the development of heterogeneous catalysts (*e.g.*, solid catalysts immobilized on nanoparticles or supports).

A significant shift toward solvent-free mechanochemical and thermal methods is evident in recent studies. Traditional methods using dichloroethane or ethyl acetate are being phased out because of their high environmental impact. These data suggest that solvent-free conditions often lead to a “rate acceleration” effect due to higher reactant concentration, which is particularly pronounced in the formation of the reactive *ortho*-quinone methide intermediate.

Substrate scope: most studies focus on simple aromatic aldehydes and amides. There is a notable lack of research regarding complex, sterically hindered, or highly deactivated substrates, which are often found in drug-like molecules.

Stereoselectivity: although AANs possess a chiral center, the vast majority of current catalytic systems are racemic. The development of asymmetric Betti reactions using chiral heterogeneous catalysts or biocatalysts represents a major frontier in the field.

Scalability: most reported yields are based on milligram-scale laboratory experiments. The transition to flow chemistry or large-scale industrial manufacturing remains largely unexplored and is a prerequisite for the commercialization of AAN-based pharmaceuticals.

Studying heterogeneous catalysts, particularly nanocatalysts, for the production of amidoalkyl naphthol (AAN) is a rapidly growing field. Heterogeneous catalysts can be readily reused and recovered, whereas homogeneous catalysts are more challenging to separate and may not be recyclable. Reusability, a large surface area, and simple separation are the main advantages of these methods. This chapter provides several examples, including AgI nanoparticles, pectin-based solid acids, and nanocrystalline lead sulfide (NCLS). One type of heterogeneous catalyst that helps increase recovery and decrease catalytic loss is the magnetic catalyst, which can be easily separated *via* magnetic force.^112^

A highly efficient and direct protocol for the synthesis of AANs via a one-pot multicomponent reaction of acetamide, β-naphthol, and aromatic aldehydes catalysed by (Fe(HSO_4_)_3_, 5 mol%) has been developed under solvent-free and microwave conditions. This method offers several benefits, including a quick reaction time, simplified work-up, high yield, catalyst reusability, and environmental friendliness. Notably, the resulting AANs can be further transformed into biologically active drug-like compounds through amide hydrolysis by converting them into 1-aminomethyl-2-naphthols (Scheme 21).^48^

**Scheme 21:**
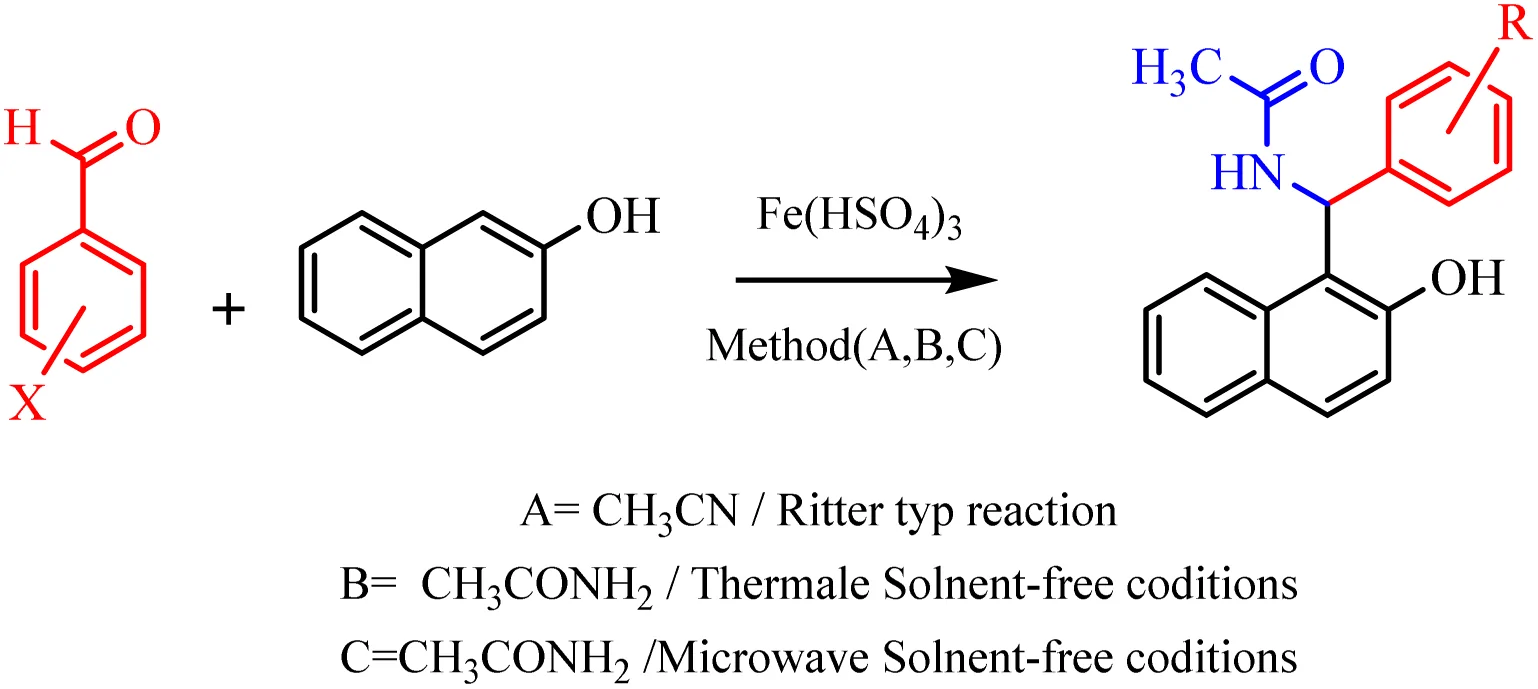
Fe(HSO_4_)_3_ as a catalyst for the easy synthesis of AANs.

An *et al.*^44^ synthesized AANs through a multicomponent reaction of 2-naphthol, aldehyde, and urea/amide catalysed by the recyclable and eco-friendly polymer-supported sulfonic acid NKC-9 as the catalyst, resulting in high yields of the desired products without the need for excess solvents (Scheme 22).

**Scheme 22:**
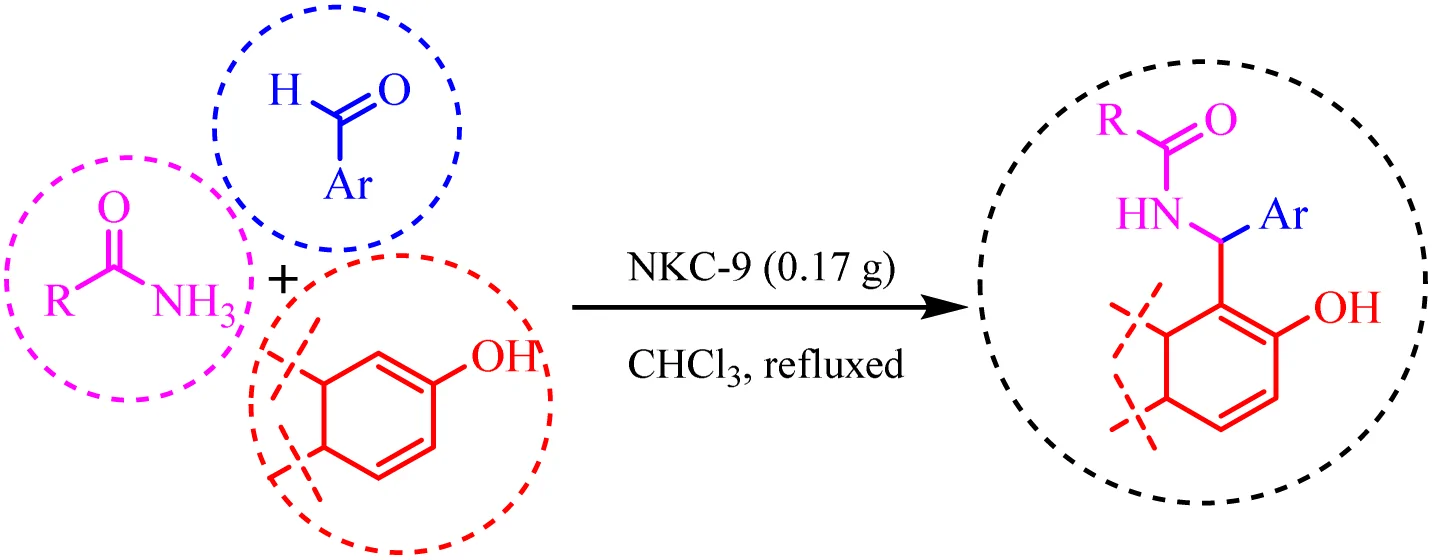
Preparation of AANs using NKC-9.

Nanocrystalline lead sulfide (PbS) was also applied to the synthesis of AANs. AANs were produced under solvent-free conditions with yields ranging from 85% to 95% using the recyclable PbS. The catalysts were evaluated via X-ray diffraction, transmission electron microscopy, and adsorption/desorption analysis (Scheme 23).^58^

**Scheme 23:**
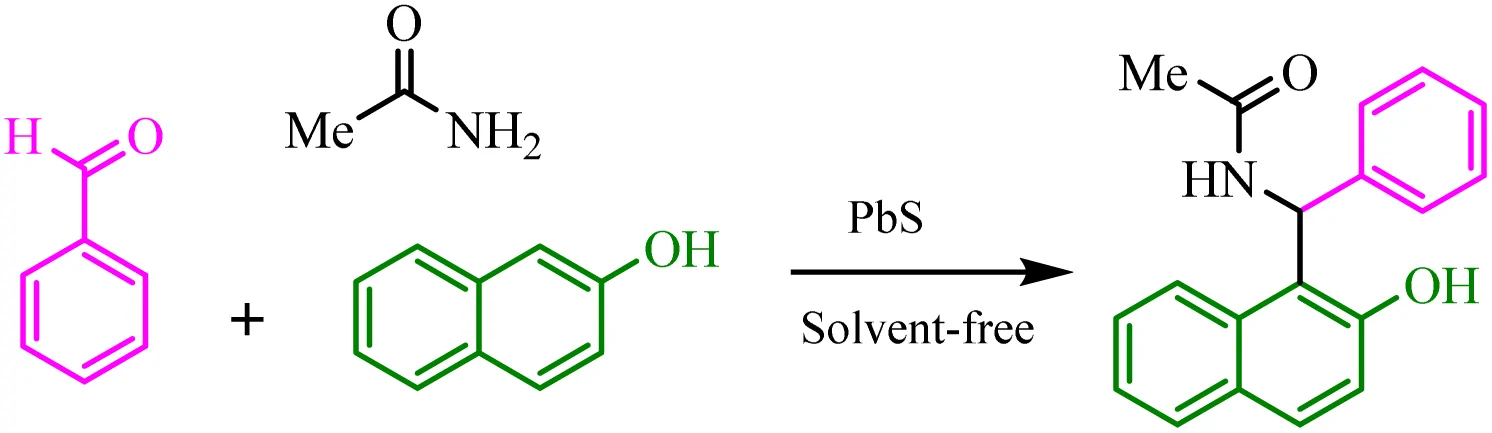
PbS as a solid catalyst for the preparation of AANs.

The one-pot three-component reaction of aromatic aldehydes, acetamide, and 2-naphthol in the presence of AgI NPs (0.3 g) as an effective heterogeneous catalyst produced the target AANs under moderate conditions. This approach has several benefits, including a high yield, short reaction time, mild reaction temperature, environmentally friendly reaction conditions, straightforward purification, and good catalyst reusability (Scheme 24).^59^

**Scheme 24:**
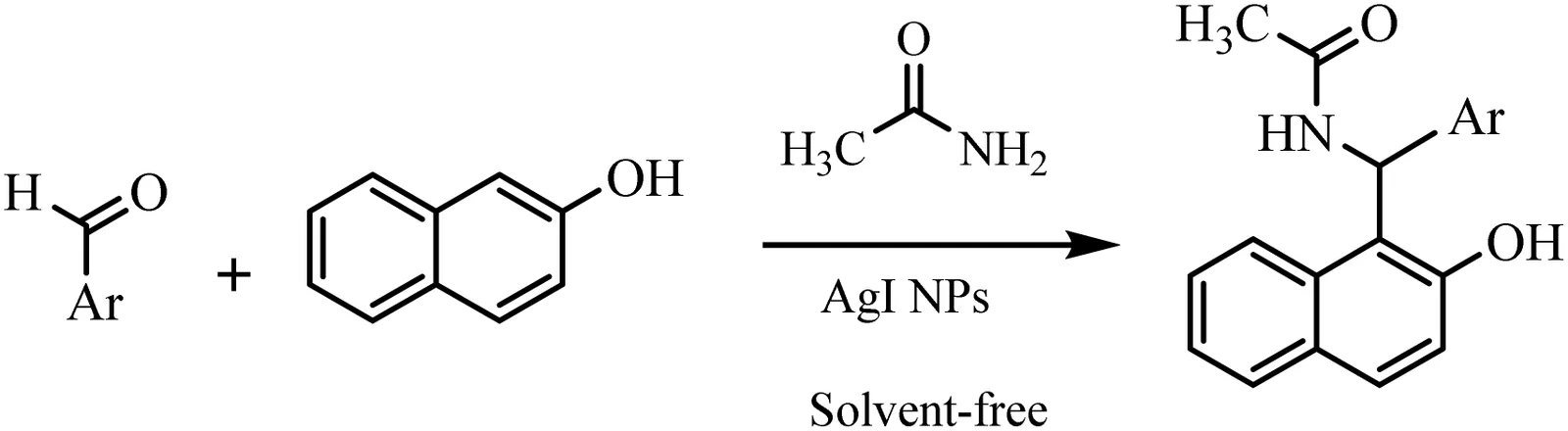
AgI NPs as nanocatalysts for the synthesis of AANs.

The widespread use of homogeneous acids raises significant environmental concerns due to their corrosive properties and the challenges associated with their recovery. In contrast, the development of heterogeneous catalysts with high Brønsted acidity and benign characteristics is challenging. As a result, pectin has been chosen as an affordable and eco-friendly support material for preparing solid acids with high Brønsted acidity. The use of oxidised pectin modified with sulfonic acid groups as a bio-derived solid acid in a multicomponent condensation synthesis of aromatic aldehydes, urea or amides, and 2-naphthol, under solvent-free conditions, as a recyclable catalyst, with high yields (Scheme 25), has been reported.^29^

**Scheme 25:**
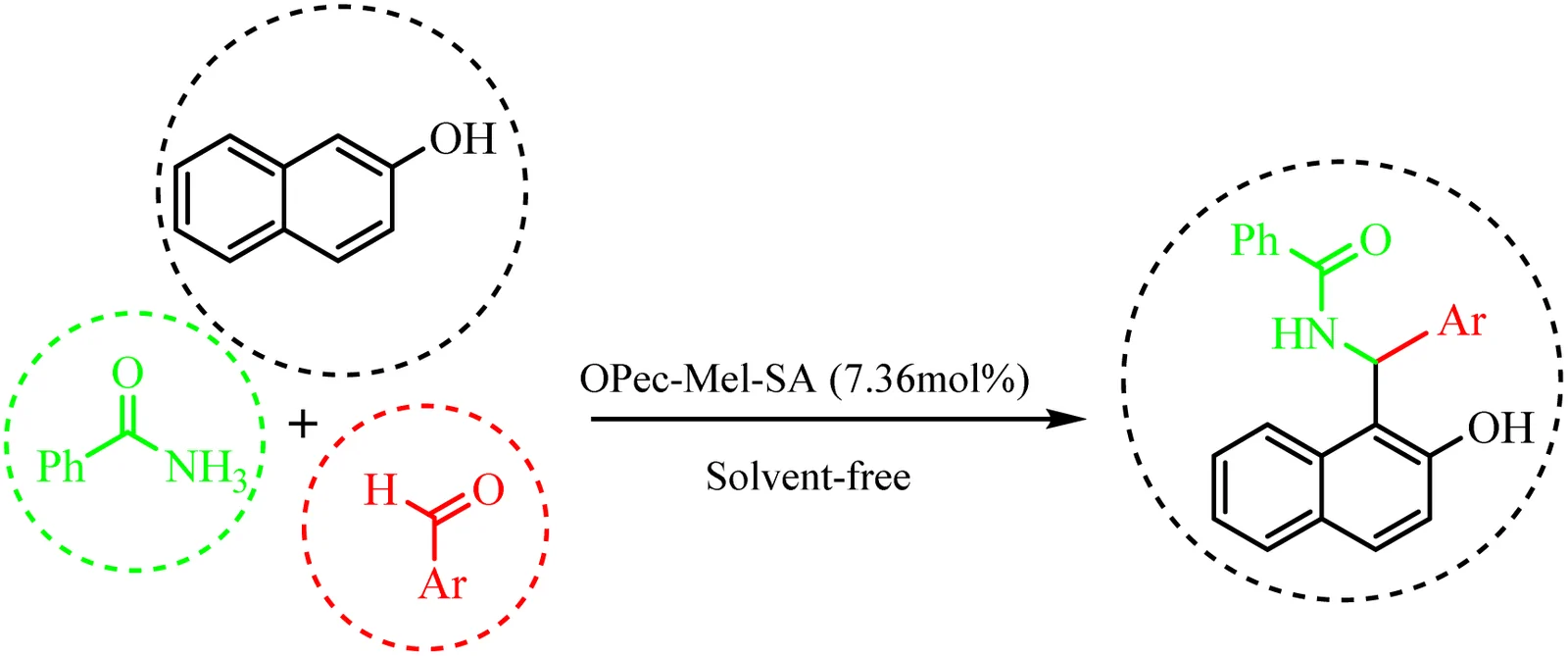
OPec-Mel-SA for AAN synthesis.

Shahbazarab and Nasr-Esfahani^30^ noted that the development of sustainable organic synthesis in the future will necessitate the utilization of renewable sources possessing desirable attributes, such as economic viability, biodegradability, abundance, and durability, along with eco-friendly raw materials, owing to the increasing demand for environmentally benign chemical processes in response to the pressing need for sustainable chemical practices in the chemical manufacturing industry (green chemistry). An example of a material displaying these favorable properties is rice husk ash (RHA), which was used as a precursor for synthesizing Fe_3_O_4_@RHA@MEL-SO_3_H nanocatalysts with solid-acidic properties *via* a controlled addition process involving Fe_3_O_4_ nanoparticles, melamine, and chlorosulfonic acid (ClSO_3_H) in distinct stages and solvents. The resulting nanocatalyst was characterized using energy-dispersive X-ray spectroscopy, Fourier-transform infrared spectroscopy, vibrating sample magnetometry (VSM), thermogravimetric analysis, field-emission scanning electron microscopy, and X-ray diffraction. Under solvent-free conditions, 2-naphthol, diverse amides, and aromatic aldehydes were used to prepare the corresponding AANs. The optimum catalyst was Fe_3_O_4_@RHA@MEL-SO_3_H (0.011 g), which allowed a short reaction time (3–42 min) and gave an excellent yield (85–92%) (Scheme 26).

**Scheme 26:**
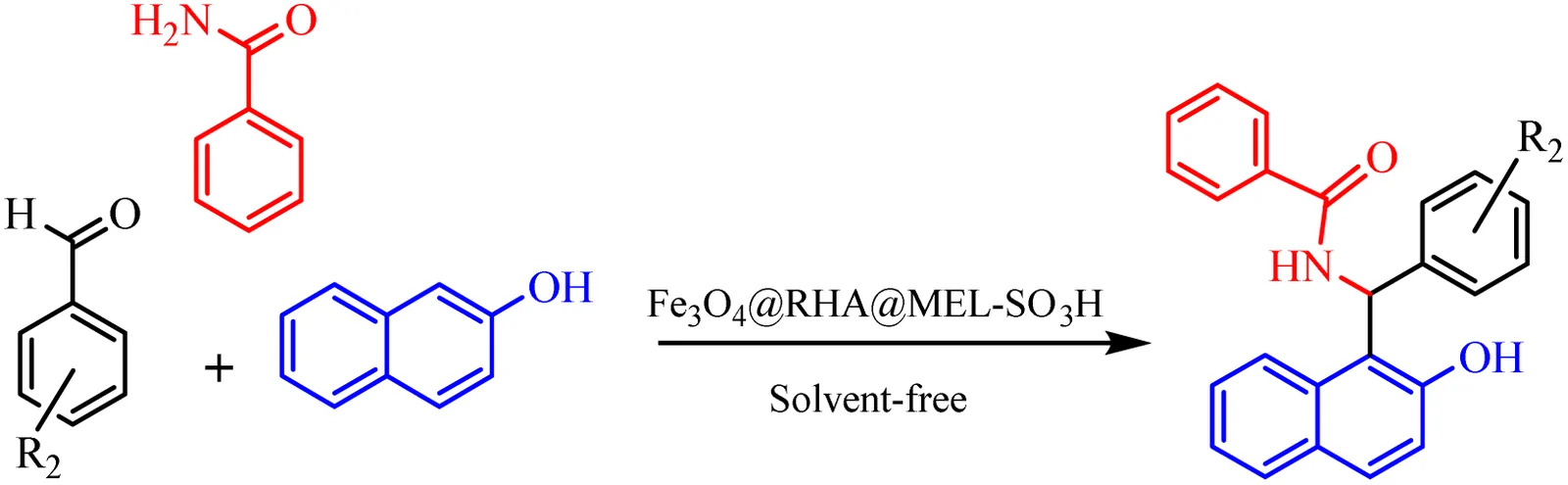
Fe_3_O_4_@RHA@MEL-SO_3_H catalyst AANs.

In another work, a simple aliphatic Schiff base ligand (-N_2_O_2_) was prepared and immobilized onto Fe_3_O_4_@SiO_2_ nanoparticles. The immobilized tetradentate Schiff base ligand underwent conversion to a Cu(ii) Schiff base complex upon reaction with a copper acetate salt. The catalytic properties of the nanoparticles were evaluated for the synthesis of AANs. Magnetic solid-base catalysts offer significant potential for facile catalyst separation *via* magnetic force, thereby minimizing catalyst loss and enhancing post-reaction recovery. The small particle size of the nanoparticles facilitates better interaction with reactants because of their increased surface area, resulting in improved yields of the reaction and enhanced reactivity properties compared with those of their bulk counterparts (Scheme 27).^31^

**Scheme 27:**
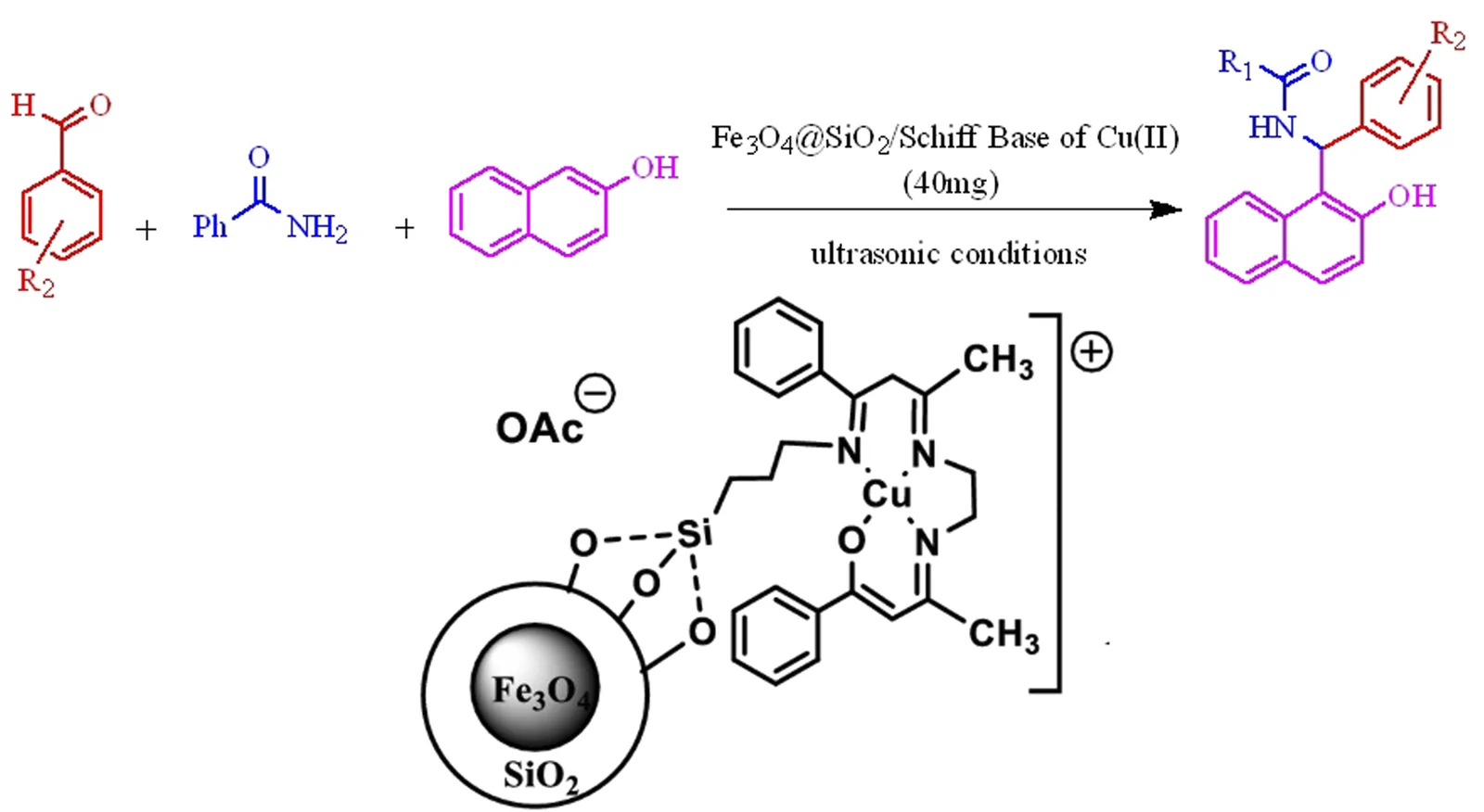
Preparation of AANs using Fe_3_O_4_@SiO_2_/Schiff base/Cu(ii).

The favorable characteristics of filamentous silica nanoparticles (KCC-1) make them promising candidates for the synthesis of heterogeneous catalysts. As a result, these compounds are considered suitable supports for catalyst preparation. In this work, CdCl_2_ was successfully immobilized onto modified KCC-1 surfaces. The reactions of 2-naphthol, acetamide, or 2-hydroxy-1,4-naphthoquinone amide with aryl aldehydes in solvents are catalysed by a small amount of KCC-1/ECH-Meg/Cd(ii) under mild conditions. This method yields amidoalkyl derivatives in high yields, with short reaction times, straightforward purification procedures, and catalyst reusability (Scheme 28).^113^

**Scheme 28:**
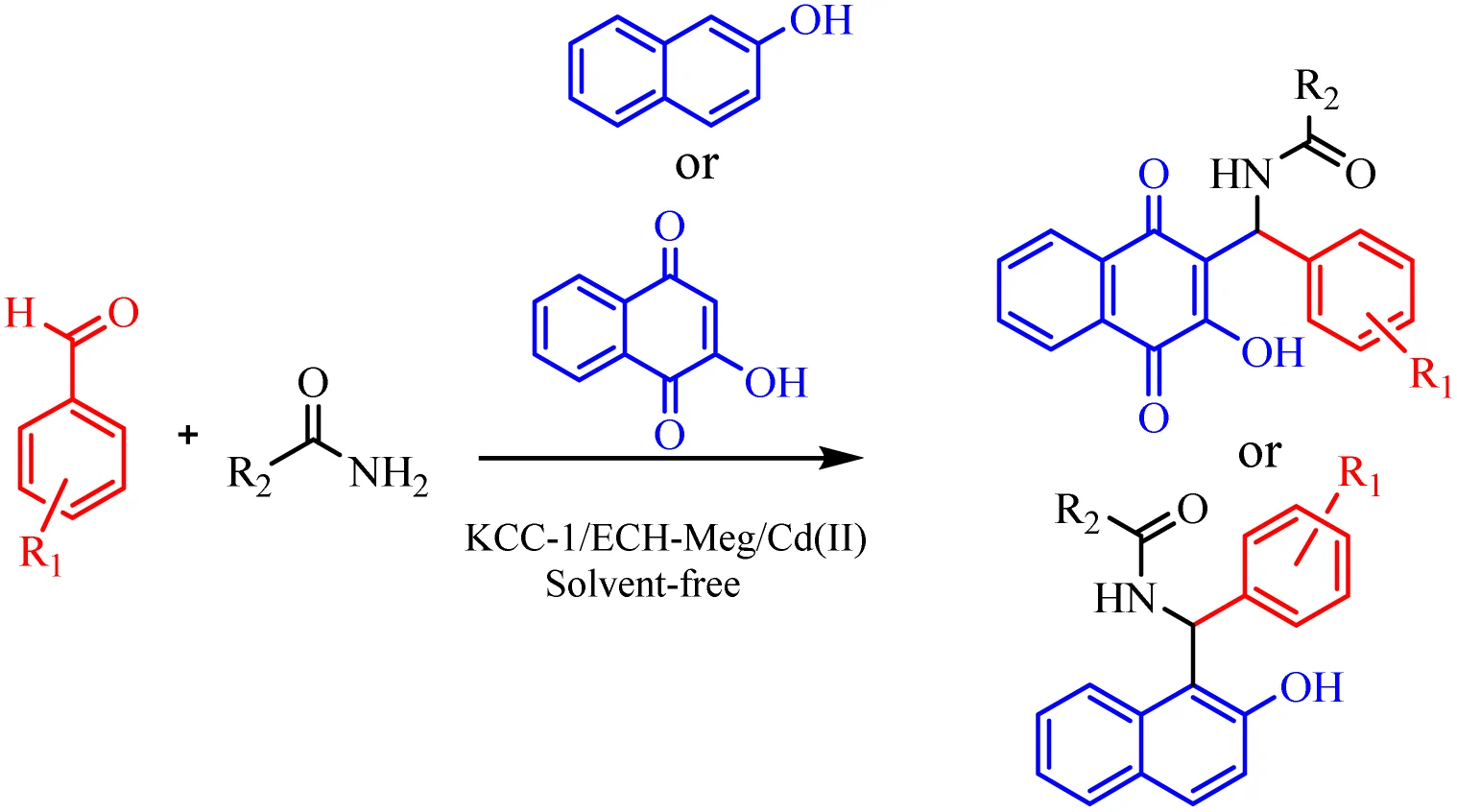
KCC-1/ECH-Meg/Cd(ii) catalyst for the preparation of AANs.

One method employs CuFe_2_O_4_/KCC-1/PMA (0.03 g) as the catalyst, along with aromatic benzaldehyde derivatives, naphthoquinone or β-naphthol, and amide compounds under solvent-free conditions. This method provides several benefits, including a short reaction time, high yields, moderate reaction temperature, easy purification, and the ability to reuse the catalyst (Scheme 29).^114^

**Scheme 29:**
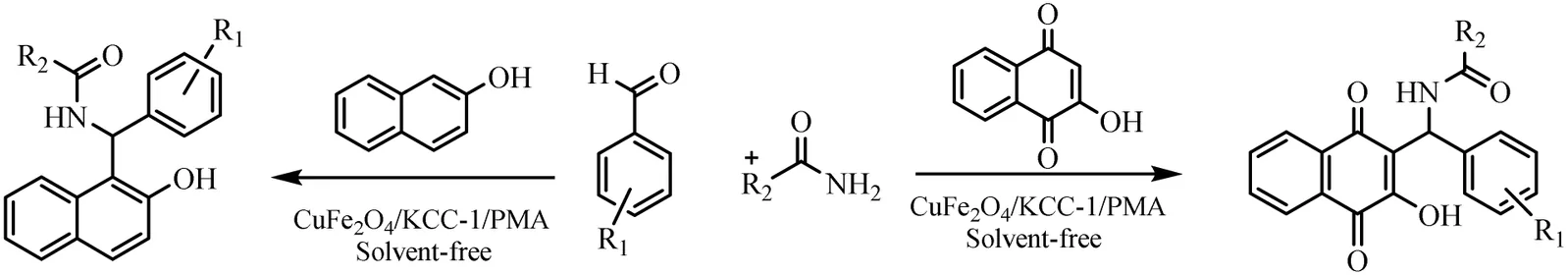
Preparation of AANs using CuFe_2_O_4_/KCC-1/PMA.

In a research work, we successfully created and employed a highly efficient catalyst for the synthesis of amidoalkyl (naphthoquinone and β-naphthol) compounds. The process of creating the catalysts involved modifying magnetic manganese oxide nanowires (M-MnO_2_ NWs) with filamentous silica nanoparticles and immobilizing phosphomolybdic acid (M-MnO_2_NW/KCC-1/PMA). This solid acid catalyst provides an easy, clean, and eco-friendly approach for the preparation of these compounds under solvent-free conditions. This method offers several benefits, such as environmentally friendly reaction conditions, shorter reaction times, and high to excellent yields of products (Scheme 30)^115^ Heterogeneous catalysts are highly valued for their straightforward separation, reusability, and exceptional durability, which renders them particularly attractive for sustainable chemical processes. Their application in multicomponent reactions has drawn considerable attention owing to improved reaction efficiency and reduced environmental impact (Table 3).

**Scheme 30:**
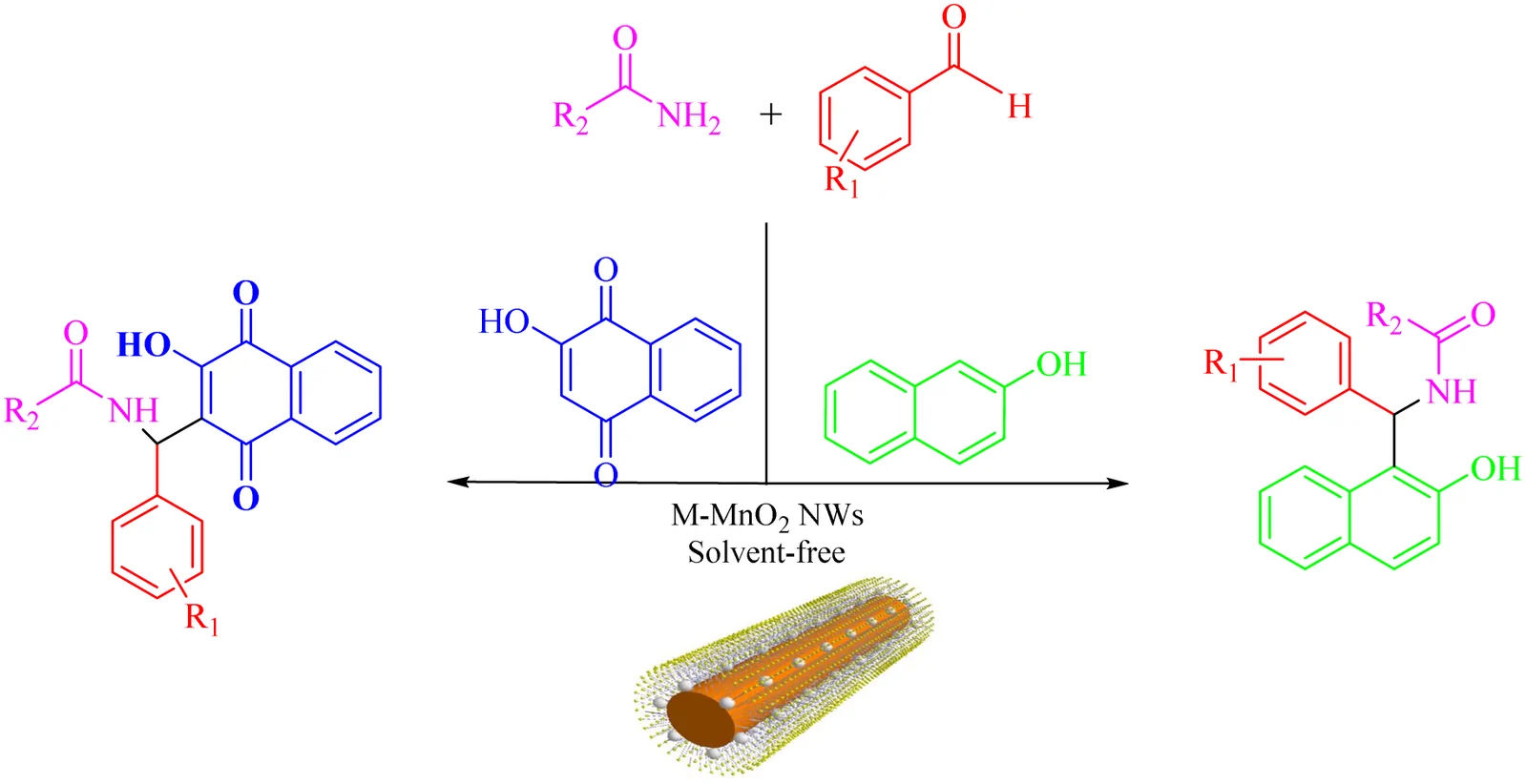
M-MnO_2_ NWs as catalysts for the synthesis of AANs.

## Conclusions

In conclusion, this comprehensive review highlights the significant progress made in the multicomponent synthesis of amidoalkyl naphthols (AANs), confirming their position as highly relevant scaffolds in medicinal chemistry. The shift toward heterogeneous catalysis, including the use of highly recyclable organocatalysts and immobilized metal complexes, has markedly improved the sustainability and step economy of AAN production. Furthermore, our analysis, which quantitatively compares catalytic systems using metrics such as atom economy and reusability, provides a clear benchmark for evaluating the greenness of established methodologies. However, the pursuit of truly sustainable and scalable AAN synthesis requires exploring disruptive methodologies that go beyond conventional heating and stirring. To propel the field toward zero-waste and ultra-efficient AAN production, future research should focus on the following emerging green technologies: (1) biocatalysis: the incorporation of enzymes (*e.g.*, lipases, esterases) or whole-cell systems offers a promising pathway for AAN synthesis under exceptionally mild conditions (ambient temperature, aqueous media, near-neutral pH). This eliminates the need for corrosive acid/base catalysts and expensive metal reagents, paving the way for green, highly selective transformations. (2) Mechanochemistry (ball milling): mechanochemical activation offers a route to synthesising AANs in a solvent-free or near-solvent-free environment. The use of mechanical energy, such as high-speed ball milling, can significantly reduce energy input and eliminate solvent waste, thereby dramatically improving the overall E-factor of the process. (3) Machine learning-guided catalyst design: the future of catalyst discovery lies in data-driven synthesis. Leveraging machine learning (ML) and artificial intelligence (AI) can drastically accelerate the identification and optimization of novel, highly efficient, and recyclable catalysts for AAN multicomponent reactions. ML algorithms can screen vast chemical spaces, predict optimal catalyst structures and reaction conditions (temperature, solvent choice), and minimize trial-and-error experimentation, ensuring the rapid development of sustainable AAN synthetic routes. By strategically adopting these forward-looking approaches, the chemical community can ensure that continued exploration of the biologically valuable AAN scaffold is conducted in accordance with the strictest principles of sustainability and environmental responsibility.

## Conflicts of interest

There are no conflicts to declare.

## Data Availability

No new data were generated or analyzed in this review article. The findings and conclusions are based on the existing literature.
